# Reactions of diphosphine-stabilized Os_3_ clusters with triphenylantimony: syntheses and structures of new antimony-containing Os_3_ clusters *via* Sb–Ph bond cleavage†

**DOI:** 10.1039/d2ra07284j

**Published:** 2023-01-18

**Authors:** Fahmida Islam, Md. Sohag Hasan, Shishir Ghosh, Michael G. Richmond, Shariff E. Kabir, Herbert W. Roesky

**Affiliations:** a Department of Chemistry, Jahangirnagar University Savar Dhaka 1342 Bangladesh sghosh_006@yahoo.com skabir_ju@yahoo.com; b Department of Chemistry, University of North Texas 1155 Union Circle, Box 305070 Denton TX 76203 USA; c Institute of Inorganic Chemistry, Georg-August University of Göttingen Tammannnstr 4 37077 Göttingen Germany hroesky@gwdg.de

## Abstract

The reactivity of the trimetallic clusters [Os_3_(CO)_10_(μ-dppm)] [dppm = bis(diphenylphosphino)methane] and [HOs_3_(CO)_8_{μ_3_-Ph_2_PCH_2_PPh(C_6_H_4_-μ_2_,σ^1^)}] with triphenylantimony (SbPh_3_) has been examined. [Os_3_(CO)_10_(μ-dppm)] reacts with SbPh_3_ in refluxing toluene to yield three new triosmium clusters [Os_3_(CO)_9_(SbPh_3_)(μ-dppm)] (1), [HOs_3_(CO)_7_(SbPh_3_){μ_3_-Ph_2_PCH_2_PPh(C_6_H_4_-μ_2_,σ^1^)}] (2), and [HOs_3_(CO)_7_(SbPh_3_)(μ-C_6_H_4_)(μ-SbPh_2_)(μ-dppm)] (3). [HOs_3_(CO)_8_{μ_3_-Ph_2_PCH_2_PPh(C_6_H_4_-μ_2_,σ^1^)}] reacts with SbPh_3_ (excess) at room temperature to afford [Os_3_(CO)_8_(SbPh_3_)(η^1^-Ph)(μ-SbPh_2_)(μ-dppm)] (4) as the sole product. A series of control experiments have also been conducted to establish the relationship between the different products. The molecular structure of each product has been determined by single-crystal X-ray diffraction analysis, and the bonding in these new clusters has been investigated by electronic structure calculations.

## Abstract 2

The reactivity of the clusters [Os_3_(CO)_10_(μ-dppm)] and [HOs_3_(CO)_8_{μ_3_-Ph_2_PCH_2_PPh(C_6_H_4_-μ_2_,σ^1^)}] with SbPh_3_ has been examined.

## Graphical abstract

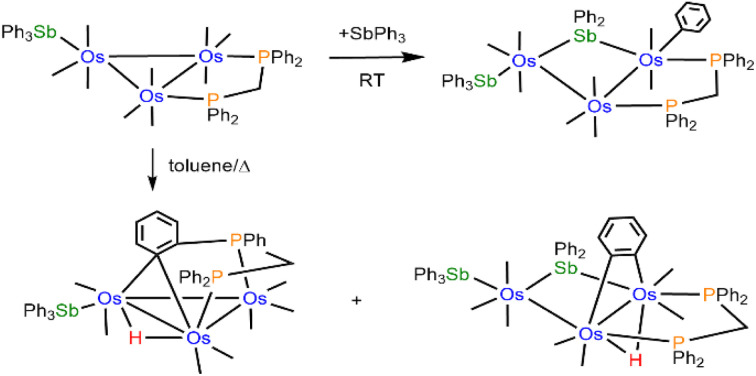

## 1. Introduction

Transition metal clusters containing oxophilic Sn, Sb, and Bi elements can serve as single-source precursors for nanoscale heterogeneous catalysts of commercial interest, especially hydrogenation, dehydrogenation, and oxidation reactions.^1–4^ A popular method to incorporate tin and antimony ligands into the coordination sphere of low-valent transition metal clusters is to use organotin hydrides (HSnR_3_) and triorganostibines (SbR_3_). The resulting organotin ligands generated by Sn–H oxidative addition of the tin reactant are strong donors and readily survive the calcination process. In comparison, metal cluster–SbR_3_ bonds are relatively weak, and the SbR_3_ ligand readily dissociates during calcinations to furnish composites with high metal : Sb ratios.^5^ As a result, the reactivity of organostibines towards low-valent transition-metal clusters remains relatively unexplored,^6–10^ although a large number of antimony-containing metal clusters have been reported over the years using Sb–Cl, Sb–H, or Sb–Sb bond cleavage reactions, as exemplified by the work of Leong and coworkers.^11–13^

We have been interested in the reactions of low-valent transition-metal clusters that contain oxophilic main group elements such as tin and antimony for over a decade.^10,14,15^ Recently, we have reported our results on several di- and trirhenium complexes containing up to three antimony donor ligands from the reactions of [Re_2_(CO)_9_(NCMe)] and [H_3_Re_3_(CO)_11_(NCMe)] with SbPh_3_.^10^ In continuation of our work on the reactivity of triorganostibines with transition metal clusters, we have examined the reactions of the diphosphine-substituted triosmium clusters [Os_3_(CO)_10_(μ-dppm)] and [HOs_3_(CO)_8_{μ_3_-Ph_2_PCH_2_PPh(C_6_H_4_-μ_2_,σ^1^)}] with SbPh_3_. We have isolated and characterized four new triosmium clusters containing Sb-based ligands from these reactions, which are discussed herein. The solid-state structures for products 1–4 have been determined by X-ray crystallography, and the bonding in these clusters has been explored by electronic structure calculations.

## 2. Experimental section

### 2.1. General remarks

All reactions were carried out under an inert atmosphere of nitrogen using standard Schlenk techniques unless otherwise noted. Reagent-grade solvents were dried by standard methods and freshly distilled before use. [Os_3_(CO)_12_] was purchased from Strem Chemical Inc. and used without further purification. Bis(diphenylphosphino)methane (dppm) and triphenylstibine (SbPh_3_) were purchased from Sigma-Aldrich and used as received. The starting clusters [Os_3_(CO)_10_(μ-dppm)] and [HOs_3_(CO)_8_{μ_3_-Ph_2_PCH_2_PPh(C_6_H_4_-μ_2_,σ^1^)}] were prepared according to the published procedures.^16^ IR spectra were recorded on a Shimadzu FTIR Prestige 21 spectrophotometer, while ^1^H and ^31^P{^1^H} NMR spectra were recorded on a Bruker Avance III HD (400 MHz) instrument. All chemical shifts are reported in ppm units and are referenced to the residual protons of the deuterated solvents (^1^H) and external 85% H_3_PO_4_ (^31^P). Elemental analyses were performed by the Microanalytical Laboratories of the Wazed Miah Science Research Center at Jahangirnagar University. All products reported herein were separated in the air by TLC plates coated with 0.25 mm silica gel (HF_254_-type 60, E. Merck, Germany).

### 2.2. Reaction of [Os_3_(CO)_10_(μ-dppm)] with SbPh_3_ at 110 °C

SbPh_3_ (57 mg, 0.16 mmol) was added to a toluene solution (30 mL) of [Os_3_(CO)_10_(μ-dppm)] (0.10 g, 0.081 mmol) and the mixture was heated at reflux for 3 h. The solution was then allowed to cool at room temperature. The solvent was removed under vacuum, and the residue was separated by TLC on silica gel. Elution with cyclohexane/CH_2_Cl_2_ (3 : 2, v/v) developed seven bands. The first and fourth bands were unreacted SbPh_3_ (trace) and [Os_3_(CO)_10_(μ-dppm)] (18 mg). The sixth, seventh, and fifth bands afforded the following compounds in order of elution: [Os_3_(CO)_9_(SbPh_3_)(μ-dppm)] (1) (36 mg, 29%) isolated as orange crystals, [HOs_3_(CO)_7_(SbPh_3_){μ_3_-Ph_2_PCH_2_PPh(C_6_H_4_-μ_2_,σ^1^)}] (2) (23 mg, 19%) isolated as green crystals, and [HOs_3_(CO)_7_(SbPh_3_)(μ-C_6_H_4_)(μ-SbPh_2_)(μ-dppm)] (3) (38 mg, 25%) isolated as yellow crystals after recrystallization from *n*-hexane/CH_2_Cl_2_ at 4 °C. The contents of the second and third bands were too small for complete characterization. Analytical and spectroscopic data for 1: anal. calcd for C_52_H_37_O_9_Os_3_P_2_Sb: C, 40.03; H, 2.39. Found: C, 40.31; H, 2.51. IR (*ν*_CO_, CH_2_Cl_2_): 2060 w, 1998 m, 1977 vs, 1958 w, 1934 w cm^−1^. ^1^H NMR (CDCl_3_): 7.58–7.31 (m, 35H), 5.01 (t, *J* 10.8 Hz, 2H). ^31^P{^1^H} NMR (CDCl_3_): −27.1 (d, *J*_PP_ 60 Hz, 1P), −29.1 (d, *J*_PP_ 60 Hz, 1P). Analytical and spectroscopic data for 2: anal. calcd for C_50_H_37_O_7_Os_3_P_2_Sb: C, 39.92; H, 2.48. Found: C, 40.20; H, 2.61. IR (*ν*_CO_, CH_2_Cl_2_): 2029 s, 1983 vs, 1956 w, 1919 m cm^−1^. ^1^H NMR (CD_2_Cl_2_): 8.32 (d, *J* 6.8 Hz, 1H), 7.99 (t, *J* 8.8 Hz, 2H), 7.76 (m, 2H), 7.65 (m, 5H), 7.58–7.46 (m, 17H), 7.06 (m, 1H), 6.99 (m, 2H), 6.59 (t, *J* 7.4 Hz, 1H), 6.31 (m, 2H), 5.99 (t, *J* 7.2 Hz, 1H), 4.97 (m, 1H), 3.87 (m, 1H), −12.51 (dd, *J*_PH_ 34.0, 11.6 Hz, 1H). ^31^P{^1^H} NMR (CDCl_3_): −17.7 (d, *J* 74 Hz, 1P), −20.9 (m, 1P). Analytical and spectroscopic data for 3: anal. calcd for C_68_H_52_O_7_Os_3_P_2_Sb_2_: C, 43.97; H, 2.82. Found: C, 44.25; H, 2.91. IR (*ν*_CO_, CH_2_Cl_2_): 2046 m, 2000 vs, 1988 sh, 1971 vs, 1938 w, 1925 w cm^−1^. ^1^H NMR (CDCl_3_): 7.43 (m, 4H), 7.30 (m, 14H), 7.17 (m, 8H), 7.08 (m, 8H), 6.95 (m, 8H), 6.80 (t, *J* 7.2 Hz, 2H), 6.73 (t, *J* 7.2 Hz, 1H), 6.33 (d, *J* 7.2 Hz, 2H), 6.23 (t, *J* 6.8 Hz, 1H), 5.85 (d, *J* 6.8 Hz, 1H), 2.28 (m, 1H), 2.05 (m, 1H), −18.70 (t, *J* 9.6 Hz, 1H). ^31^P{^1^H} NMR (CDCl_3_): 11.9 (d, *J*_PP_ 55 Hz, 1P), 2.2 (d, *J*_PP_ 55 Hz, 1P).

### 2.3. Reaction of [Os_3_(CO)_10_(μ-dppm)] with SbPh_3_ in the presence of Me_3_NO at room temperature

To a CH_2_Cl_2_ solution (15 mL) of [Os_3_(CO)_10_(μ-dppm)] (0.10 g, 0.081 mmol) and SbPh_3_ (43 mg, 0.12 mmol) was added Me_3_NO (11 mg, 0.15 mmol), and the reaction mixture was stirred at room temperature for 5 h. The solvent was removed under reduced pressure and the crude product was chromatographed by TLC on silica gel. Elution with cyclohexane/CH_2_Cl_2_ (3 : 2 v/v) developed six bands. The first and second bands were SbPh_3_ (trace) and [Os_3_(CO)_10_(μ-dppm)] (3 mg), while the third and fifth bands afforded 1 (60 mg, 48%) and 3 (5.0 mg, 3.0%). The contents of the other bands were too small for complete characterization.

### 2.4. Thermolysis of [Os_3_(CO)_9_(SbPh_3_)(μ-dppm)] (1) at 110 °C

A toluene solution (15 mL) of 1 (30 mg, 0.019 mmol) was heated at 110 °C for 2.5 h. Upon cooling, the solvent was removed by rotary evaporation, after which the residue was separated by TLC on silica gel. Elution with cyclohexane/CH_2_Cl_2_ (3 : 2 v/v) developed four bands. The first and second band gave [HOs_3_(CO)_8_{μ_3_-Ph_2_PCH_2_PPh(C_6_H_4_-μ_2_,σ^1^)}] (5.0 mg, 22%) and [HOs_3_(CO)_7_(SbPh_3_){μ_3_-Ph_2_PCH_2_PPh(C_6_H_4_-μ_2_,σ^1^)}] (2) (18 mg, 62%), both of were isolated as green crystals after recrystallization from *n*-hexane/CH_2_Cl_2_ at 4 °C. The contents of the other bands were too small for complete characterization.

### 2.5. Reaction of [HOs_3_(CO)_8_{μ_3_-Ph_2_PCH_2_PPh(C_6_H_4_-μ_2_,σ^1^)}] with SbPh_3_

SbPh_3_ (30 mg, 0.085 mmol) was added to a CH_2_Cl_2_ (15 mL) solution of [HOs_3_(CO)_8_{μ_3_-Ph_2_PCH_2_PPh(C_6_H_4_-μ_2_,σ^1^)}] (50 mg, 0.042 mmol) and the mixture was stirred at room temperature for 28 h. The initial green-colored solution slowly turned a light yellow-orange in color by the end of the reaction. The solvent was removed by rotary evaporation under reduced pressure, and the residue was subjected to TLC on silica gel. Elution with cyclohexane/CH_2_Cl_2_ (7 : 3, v/v) developed three bands. The first band was unreacted [HOs_3_(CO)_8_{μ_3_-Ph_2_PCH_2_PPh(C_6_H_4_-μ_2_,σ^1^)}] (10 mg) and the third band furnished [Os_3_(CO)_8_(SbPh_3_)(η^1^-Ph)(μ-SbPh_2_)(μ-dppm)] (4) (61 mg, 75%), which was isolated as orange crystals after recrystallization from ethanol/acetone at room temperature. The contents of the second band were too small for complete characterization. Analytical and spectroscopic data for 4: anal. calcd for C_69_H_52_O_8_Os_3_P_2_Sb_2_: C, 43.96; H, 2.78. Found: C, 44.12; H, 2.87. IR (*ν*_CO_, CH_2_Cl_2_): 2052 w, 2012 w, 1985 vs, 1969 s, 1935 m, 1921 m, 1900 w cm^−1^. ^1^H NMR (CD_2_Cl_2_): aromatic region: both isomers: 7.63 (m, 5H), 7.57 (m, 9H), 7.45 (m, 2H), 7.39 (m, 9H), 7.31 (m, 10H), 7.21 (m, 6H), 7.14 (m, 4H), 6.90 (m, 1H), 6.67 (m, 3H); aliphatic region: major isomer: 4.01 (t, *J* 10.4 Hz, 2H), minor isomer: 3.94 (t, *J* 10.4 Hz). ^31^P{^1^H} NMR (CD_2_Cl_2_): major isomer: 13.8 (*J*_PP_ 66 Hz), 0.2 (d, *J*_PP_ 66 Hz, 1P); minor isomer: 15.1 (d, *J*_PP_ 66 Hz, 1P), 1.6 (d, *J*_PP_ 66 Hz, 1P).

### 2.6. Thermolysis of [Os_3_(CO)_8_(SbPh_3_)(η^1^-Ph)(μ-SbPh_2_)(μ-dppm)] (4)

A toluene solution (15 mL) of 4 (30 mg, 0.016 mmol) was heated at reflux for 3 h, after which the solution was then allowed to cool at room temperature. The solvent was removed under reduced pressure and the residue was purified by TLC chromatography over silica gel. Elution with cyclohexane/CH_2_Cl_2_ (7 : 3 v/v) developed four bands. The second band afforded [HOs_3_(CO)_7_(SbPh_3_){μ_3_-Ph_2_PCH_2_PPh(C_6_H_4_-μ_2_,σ^1^)}] (2) (15 mg, 63%) and third band yielded [HOs_3_(CO)_7_(SbPh_3_)(μ-C_6_H_4_)(μ-SbPh_2_)(μ-dppm)] (3) (9 mg, 30%). The contents of the first and fourth bands were too small for complete characterization.

### 2.7. Reaction of [HOs_3_(CO)_7_(SbPh_3_){μ_3_-Ph_2_PCH_2_PPh(C_6_H_4_-μ_2_,σ^1^)}] (2) with SbPh_3_: formation of 4

To a CH_2_Cl_2_ solution (10 mL) of 2 (10 mg, 0.007 mmol) was added SbPh_3_ (3 mg, 0.007 mmol) and the solution was stirred at 25 °C for 12 h during which time the color changed from green to yellow. The solvent was removed under reduced pressure and the residue chromatographed by TLC on silica gel. Elution with cyclohexane/CH_2_Cl_2_ (7 : 3, v/v) gave two bands. The faster-moving band gave unreacted ligand and the second band afforded [Os_3_(CO)_8_(SbPh_3_)(η^1^-Ph)(μ-SbPh_2_)(μ-dppm)] (4) (8 mg, 95%). The identity of compound 4 was confirmed by IR.

### 2.8. Thermolysis of [Os_3_(CO)_7_(SbPh_3_)(μ-C_6_H_4_)(μ-SbPh_2_)(μ-dppm)] (3)

A toluene solution (5 mL) of 3 (5 mg, 0.003 mmol) was heated at 110 °C for 3 h. The solvent was removed by rotary evaporation and the residue separated by TLC on silica gel. Elution with cyclohexane/CH_2_Cl_2_ (3 : 2, v/v) gave four bands. The first band gave unconsumed 3 and the second band afforded [Os_3_(CO)_8_(SbPh_3_)(η^1^-Ph)(μ-SbPh_2_)(μ-dppm)] (4) (3 mg, 50%). The contents of other bands were too small for complete characterization.

### 2.9. Crystal structure determinations

Single crystals of 1–4 suitable for X-ray diffraction study were grown by slow diffusion of *n*-hexane into a CH_2_Cl_2_ solution containing each compound. Suitable crystals were mounted on a Bruker D8 Venture diffractometer equipped with a PHOTON II CPAD detector using a Nylon loop and Paratone oil. The diffraction data were collected at 193(1) K for 4 and 230(1) K for clusters 1, 2, and 3 using Mo–Kα radiation (*λ* = 0.71073). Data reduction and integration were carried out with SAINT^+^,^17^ and absorption corrections were applied using the program SADABS.^18^ The structures were solved with the ShelXT^19^ structure solution program using intrinsic phasing and refined with the XL^20^ refinement package using least-squares minimization within the OLEX2 (ref. 21) graphical user interface. All non-hydrogen atoms were refined anisotropically, and the hydrogen atoms were included using a riding model. Pertinent crystallographic parameters are given in Table 1, and selected bond distances and bond angles for clusters 1–4 may be found in Table S1 (ESI).†

**Table 1:** Crystal data and structure refinement details for compounds 1–4

Compound	1	2	3	4
CCDC	2152363	2152364	2152366	2152367
Empirical formula	C_52_H_37_O_9_Os_3_P_2_Sb	C_50_H_37_O_7_Os_3_P_2_Sb·CH_2_Cl_2_	C_68_H_52_O_7_Os_3_P_2_Sb_2_	C_69_H_52_O_8_Os_3_P_2_Sb_2_
Formula weight	1560.10	1589.01	1857.13	1885.14
Temperature (K)	210(1)	210(1)	210(1)	193(1)
Wavelength (Å)	0.71073	0.71073	0.71073	0.71073
Crystal system	Monoclinic	Monoclinic	Orthorhombic	Triclinic
Space group	*C*2/*c*	*P*2_1_/*c*	*P*2_1_2_1_2_1_	*P*1̄

**Unit cell dimensions**				
*a* (Å)	42.6814(13)	19.6929(10)	15.1736(4)	11.9842(5)
*b* (Å)	11.4302(3)	13.9599(7)	20.3459(6)	12.2615(4)
*c* (Å)	19.9930(6)	20.1424(10)	21.9689(6)	22.7266(9)
*α* (°)	90	90	90	101.908(2)
*β* (°)	90.434(2)	113.6390(10)	90	98.475(2)
*γ* (°)	90	90	90	94.176(2)
Volume (Å^3^)	9753.4(5)	5072.7(4)	6782.3(3)	3213.6(2)
*Z*	8	4	4	2
Density (calculated) (mg m^−3^)	2.125	2.081	1.819	1.948
Absorption coefficient (mm^−1^)	8.461	8.235	6.482	6.843
*F*(000)	5840	2976	3504	1780
Crystal size (mm^3^)	0.321 × 0.188 × 0.127	0.221 × 0.188 × 0.079	0.398 × 0.305 × 0.108	0.225 × 0.108 × 0.038
2*θ* range for data collection (°)	4.486 to 54.48	4.516 to 56.762	4.412 to 54.466	4.458 to 56.692
Index ranges	−54 ≤ *h* ≤ 54, −14 ≤ *k* ≤ 14, −25 ≤ *l* ≤ 25	−26 ≤ *h* ≤ 26, −18 ≤ *k* ≤ 18, −26 ≤ *l* ≤ 26	−19 ≤ *h* ≤ 19, −26 ≤ *k* ≤ 26, −28 ≤ *l* ≤ 28	−15 ≤ *h* ≤ 15, −16 ≤ *k* ≤ 16, −30 ≤ *l* ≤ 30
Reflections collected	144 691	115 314	197 248	74 004
Independent reflections [*R*_int_]	10 838 [*R*_int_ = 0.0548]	12 614 [*R*_int_ = 0.0611]	15 130 [*R*_int_ = 0.0490]	15 929 [*R*_int_ = 0.0447]
Data/restraints/parameters	10 838/0/604	12 614/0/599	15 130/0/743	15 929/0/757
Goodness of fit on *F*^2^	1.029	1.025	1.116	1.026
Final *R* indices [*I* > 2*σ*(*I*)]	*R*_1_ = 0.0215, w*R*_2_ = 0.0448	*R*_1_ = 0.0290, w*R*_2_ = 0.0498	*R*_1_ = 0.0265, w*R*_2_ = 0.0596	*R*_1_ = 0.0298, w*R*_2_ = 0.0589
*R* indices (all data)	*R*_1_ = 0.0296, w*R*_2_ = 0.0479	*R*_1_ = 0.0522, w*R*_2_ = 0.0559	*R*_1_ = 0.0286, w*R*_2_ = 0.0603	*R*_1_ = 0.0482, w*R*_2_ = 0.0642
Largest diff. peak and hole (e Å^−3^)	0.79 and −0.93	1.13 and −1.05	0.98 and −1.39	1.14 and −0.90

### 2.10. Computational modeling details

All calculations were performed with the hybrid meta exchange–correlation functional M06,^22^ as implemented by the Gaussian 09 program package.^23^ The osmium and antimony atoms were described by Stuttgart–Dresden effective core potentials (ECP) and an SDD basis set,^24^ while a 6-31G(d′) basis set was employed for the remaining atoms.^25^ All calculations included Grimme's dispersion correction.^26^

The input data for the optimizations were taken from the coordinates of the experimental structures, and the Hessian matrix for each geometry-optimized structure displayed only positive eigenvalues. The natural charges (*Q*) and Wiberg bond indices (WBIs) were computed using Weinhold's natural bond orbital (NBO) program (NBO version 3.1).^27,28^Table 2 summarizes the NBO data. The geometry-optimized structures presented here have been drawn with the JIMP2 molecular visualization and manipulation program.^29^

**Table 2:** Selected natural charges (*Q*) and Wiberg bond indices (WBIs) for the DFT-optimized structures based on clusters 1–4^a^

Compound	Os_3_(CO)_10_(dppm)	A	B	C	D
**Natural charge (*Q*)**					
Os_1_	−1.45	−1.44	−1.38	−1.62	−1.60
Os_2_	−1.45	−1.49	−1.49	−1.18	−1.50
Os_3_	−1.30	−1.59	−1.19	−1.94	−1.94
Sb_1_		1.89	1.83	1.88	1.89
Sb_2_				1.67	1.72
P_1_	1.46	1.40	1.41	1.45	1.42
P_2_	1.45	1.40	1.44	1.43	1.44
H(hydride)			0.13	0.07	
C(σ-metalated)					−0.09
C(benzylidene)			−0.19		
C(benzyne1)				−0.15	
C(benzyne2)				−0.17	

**Wiberg bond index (WBI)**					
Os_1_–Os_2_	0.41	0.42	0.46	0.18	0.35
Os_2_–Os_3_	0.41	0.43	0.49	0.41	0.38
Os_1_–Os_3_	0.41	0.47	0.35	0.03	0.04
Os_3_–Sb_1_		0.80	0.76	0.81	0.79
Os_1_–Sb_2_				0.74	0.74
Os_3_–Sb_2_				0.73	0.72
Os_1_–P_1_	0.78	0.79	0.79	0.79	0.81
Os_2_–P_2_	0.80	0.80	0.78	0.79	0.81
Os_1_–C_σ-metalated_					0.76
Os_1_–C_benzylidene_			0.56		
Os_3_–C_benzylidene_			0.32		
Os_1_–H			0.45	0.40	
Os_3_–H			0.31	0.37	

a Atom-numbering sequence based on the structures depicted below:
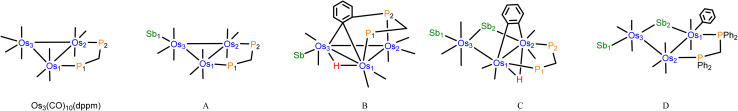

## 3. Results

### 3.1. Reaction of [Os_3_(CO)_10_(μ-dppm)] with SbPh_3_

[Os_3_(CO)_10_(μ-dppm)] reacts with SbPh_3_ (2 equiv.) in refluxing toluene to give the new clusters [Os_3_(CO)_9_(SbPh_3_)(μ-dppm)] (1), [HOs_3_(CO)_7_(SbPh_3_){μ_3_-Ph_2_PCH_2_PPh(C_6_H_4_-μ_2_,σ^1^)}] (2), and [HOs_3_(CO)_7_(SbPh_3_)(μ-C_6_H_4_)(μ-SbPh_2_)(μ-dppm)] (3) in a combined yield of 73%, after chromatographic separation and recrystallization. The three new products have been characterized by analytical and spectroscopic methods, and their molecular structures have been determined by single-crystal X-ray diffraction analyses.

**Fig. 1:**
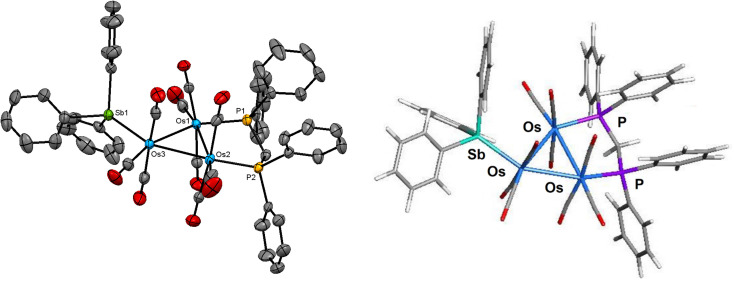
Molecular structure (left, 1) and M06-optimized structure (right, species A) of [Os_3_(CO)_9_(SbPh_3_)(μ-dppm)] showing 50% probability atomic displacement ellipsoids. Hydrogen atoms in the crystallographic structure are omitted for clarity.

The molecular structure of 1 is depicted in Fig. 1. The product contains a triosmium core ligated by nine carbonyls, and dppm and SbPh_3_ ligands. The three pnictogen donors lie in the equatorial plane defined by the three osmium atoms with the gross structural features of 1 similar to those of the structurally related phosphine-substituted triosmium clusters [Os_3_(CO)_9_(PR_3_)(μ-dppm)] [PR_3_ = PPh_3_, P(C_4_H_3_S)_3_, PPh_2_H].^30,31^ The bridging diphosphine ligates the Os(1) and Os(2) atoms, while the SbPh_3_ ligand is bonded to the Os(3) atom. The Os–P [mean 2.3299 Å] and Os–Sb [2.5921(3) Å] bond distances are similar to those distances reported in the literature for related clusters.^6,11,12^ The SbPh_3_ substitution has no effect on the Os_3_-triangle as the average Os–Os distance in 1 (2.887 Å) is identical to the Os–Os bond distances in [Os_3_(CO)_10_(μ-dppm)] (2.885 Å).^32^ The solution spectroscopic data of 1 are in accord with the solid-state structure. The ^31^P{^1^H} NMR spectrum displays two doublets at −27.1 and −29.1 ppm (*J*_PP_ 60 Hz) for the two inequivalent dppm phosphorus atoms, while the ^1^H NMR spectrum reveals a virtual triplet at 5.01 ppm (*J* 10.8 Hz) attributed to the methylene protons of the dppm ligand together with a series of aromatic multiplets due to phenyl protons of the SbPh_3_ and dppm ligands.

The bonding in 1 was investigated by DFT. The M06-optimized structure of species A is depicted alongside the solid-state structure in Fig. 1. Excellent agreement between the two structures is noted. The three osmium atoms exhibit a negative charge that ranges from −1.44 [Os(2)] to −1.59 [Os(3)] and display a mean *Q* value (measure of charge) of −1.51. The osmium charges are unremarkable compared to those values reported by us for related osmium clusters.^14*c*,33^ The charge on the antimony atom is 1.89 and is similar in magnitude to that reported for the related cluster [Ru_3_(CO)_9_(SbPh_3_)(μ-dppm)].^34^ The mean Wiberg bond index (WBI), which serves a measure of bond strength, for the three Os–Os bonds is 0.44, 0.80 for the Os–Sb bond, and 0.80 for the two Os–P bonds in A. These values are comparable in magnitude to other polynuclear osmium clusters investigated by us.^14*c*,33^ We also optimized the cluster with an axial SbPh_3_ ligand (species A_alt; not shown) and confirmed the thermodynamic preference for A, which is more stable by 2.4 kcal mol^−1^ (Δ*G*).

**Fig. 2:**
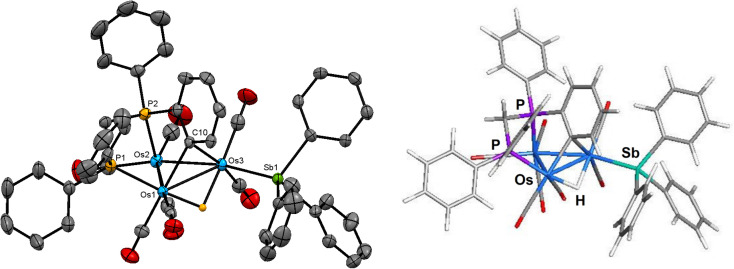
Molecular structure (left, 2) and M06-optimized structure (right, species B) of [HOs_3_(CO)_7_(SbPh_3_){μ_3_-Ph_2_PCH_2_PPh(C_6_H_4_-μ_2_,σ^1^)}] showing 50% probability atomic displacement ellipsoids. Hydrogen atoms, except the hydride, are omitted for clarity in the crystallographic structure.

The molecular structure of compound 2 is depicted in Fig. 2. The overall structure of 2 is similar to that of [HOs_3_(CO)_8_{μ_3_-Ph_2_PCH_2_PPh(C_6_H_4_-μ_2_,σ^1^)}],^16^ which is prepared from [Os_3_(CO)_10_(μ-dppm)] *via* decarbonylation at 110 °C. The cluster core consists of an approximate isosceles triangle of osmium atoms where the Os–Os bond distances range from 2.7720(3) Å [Os(1)–Os(3)] to 2.8249(3) Å [Os(1)–Os(2)]. The metalated phenyl group, which may be viewed as a benzylidene moiety, asymmetrically bridges the shortest Os–Os bond [Os(1)–Os(3) 2.7720(3) Å] using the C(10) atom [Os(1)–C(10) 2.232(4) Å and Os(3)–C(10) 2.399(4) Å]. The phosphorus atom associated with the metalated aryl ring occupies an axial coordination site to facilitate this orthometalation. The hydride ligand, which was located and refined crystallographically, spans the same Os–Os vector as the benzylidene-bridged Os(1)–Os(3) edge except that it lies below the metallic polyhedron opposite the bridging benzylidene ligand. The SbPh_3_ ligand is bound to Os(3) atom and occupies the equatorial site *trans* to the Os(2) atom. The mean Os–P bond distance in 2 (2.3302 Å) is almost identical to that found in [HOs_3_(CO)_8_{μ_3_-Ph_2_PCH_2_PPh(C_6_H_4_-μ_2_,σ^1^)}]^16^ (2.3265 Å), while the Os–Sb bond distance of 2.6343(4) Å is similar in magnitude to that observed in 1 and other related clusters.^6,11,12^

The optimized structure of species B is shown to the right of cluster 2 in Fig. 2. All of the important structural features in 2 are reproduced in species B. The *Q* values for the three osmium atoms range from −1.19 [Os(3)] to −1.49 [Os(2)], with a mean value of −1.35. The pnictogen atoms exhibit a positive charge of 0.79 (mean) and 1.83 for the P and Sb atoms, respectively. The *Q* values for the bridging hydride (0.13) and benzylidene carbon (−0.19) are consistent with earlier values reported by us for related clusters.^35^ The mean Wiberg bond index for the Os–Os bonds is 0.43, supporting the single-bond designation ascribed to these bonds in cluster 2. The WBIs for the Os–P [0.79 mean] and Os–Sb [0.76] bonds are unremarkable compared to species A. The asymmetry observed in the bridging of the Os(1)–Os(3) vector by the benzylidene and hydride ligands in 2 is reproduced in B. This trend is attributed to the ancillary SbPh_3_ group, which lengthens (weakens) the proximal Os_3_–C(benzylidene) and Os_3_–H bonds relative to their distal Os_1_–C(benzylidene) and Os_1_–H counterparts. The larger (stronger) WBI for the Os–C(benzylidene) (0.56 *versus* 0.32) and the Os–H (0.45 *versus* 0.31) bonds are associated with the Os(1) atom.^36^

The solution spectroscopic data of 2 indicate that the solid-state structure persists in solution. The IR spectrum exhibits four absorption bands between 2029–1919 cm^−1^, while the ^31^P{^1^H} NMR spectrum shows a doublet at −17.7 ppm (*J* 74 Hz) and a multiplet at −20.9 ppm for the inequivalent phosphorus atoms of the diphosphine ligand. In addition to a series of multiplets in the aromatic region for the phenyl protons, the ^1^H NMR spectrum also displays two multiplets at 4.97 and 3.87 ppm attributed to the methylene protons of the diphosphine ligand, and the upfield doublet of doublets at −12.51 ppm (*J*_PH_ 34.0, 11.6 Hz) for the bridging hydride consistent with the solid-state structure.

**Fig. 3:**
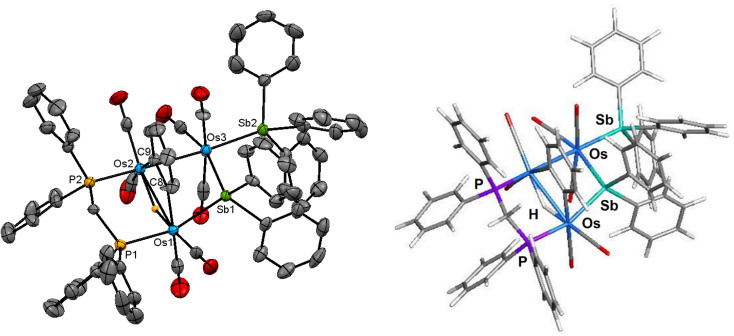
Molecular structure (left, 3) and M06-optimized structure (right, species C) of [HOs_3_(CO)_7_(SbPh_3_)(μ-C_6_H_4_)(μ-SbPh_2_)(μ-dppm)] showing 50% probability atomic displacement ellipsoids. Hydrogen atoms, except the hydride, are omitted for clarity in the crystallographic structure.

The ORTEP diagram in Fig. 3 shows the molecular structure of 3. The molecule contains 50 valence electrons and exhibits an expanded metallic polyhedron with two Os–Os single bonds [Os(1)–Os(2) 3.1729(4) Å; Os(2)–Os(3) 2.9725(4) Å]. The >4.30 Å internuclear Os(1)⋯Os(3) distance precludes any significant bonding interaction between these osmium atoms. The μ_2_-stibene moiety [Sb(1) atom], which serves as a 3e donor, tethers the two non-bonding Os(1) and Os(3) centers. The Sb(2) atom associated with the SbPh_3_ ligand is bound by the Os(3) atom and functions as a 2e ligand. The bridging dppm, C_6_H_4_ (benzyne), and hydride ligands all span the Os(1)–Os(2) vector and collectively serve to donate 5e to the total electron count. The μ-C_6_H_4_ ligand is bound to the Os(1) and Os(2) atoms *via* the C(8) and C(9) carbon atoms, respectively, and the plane containing the benzyne ligand is almost perpendicular to the Os_3_ plane based on the dihedral angle of *ca.* 81°. This bonding mode exhibited by the benzyne ligand is unremarkable in comparison to the triosmium clusters [H_3_Os_3_(CO)_8_(μ,η^2^-HC=NC_6_H_5_)(μ-C_6_H_4_)]^37^ and [HOs_3_(CO)_9_(L)(μ-SbPh_2_)(μ-C_6_H_4_)] [L = PPh_3_, PMe_2_Ph, P(C_6_H_4_Me-*o*)_3_].^8*b*^ The Os–C(benzyne) bond distances of 2.134(7) Å [Os(1)–C(8)] and 2.128(7) Å [Os(2)–C(9)] in 3 are similar in magnitude to those distances reported for [HOs_3_(CO)_9_(PPh_3_)(μ-SbPh_2_)(μ-C_6_H_4_)] [2.123(10) Å and 2.159(9) Å]^8*b*^ and [H_3_Os_3_(CO)_8_(μ,η^2^-HC=NC_6_H_5_)(μ-C_6_H_4_)] [2.133(7) Å and 2.145(6) Å].^37^ The non-bonding Os(1)⋯Os(3) edge is asymmetrically bridged by the SbPh_2_ ligand [Os(1)–Sb(1) 2.6400(6) Å and Os(3)–Sb(1) 2.7081(5) Å], and the SbPh_3_ ligand is coordinated to Os(3) [Os(3)–Sb(2) 2.6137(6) Å], occupying an equatorial site *cis* to the bridging SbPh_2_ ligand.

The M06-optimized structure of species C appears to the right of the X-ray diffraction structure of cluster 3. The *Q* values for C parallel those data reported for species A and B. The charges on the osmium atoms range from −1.18 [Os(2)] to −1.94 [Os(3)], with an average *Q* value of −1.58. The charge on the antimony atoms is sensitive to the nature of the ligand, with the stibine [Sb(2) = 1.88] donor *ca.* 11% larger than the stibene [Sb(1) = 1.67] bridging moiety. The computed Sb charges for species C are similar in magnitude to those values recently reported by us for a series of ruthenium clusters containing stibine and stibene ligands.^34^ The bridging hydride is essentially neutral based on a *Q* value of 0.07, while the mean *Q* value for the metalated benzyne carbons is −0.16. The mean charge for P(1) and P(2) atoms is 1.44. The mean WBI for the two Os–Os bonds is 0.30, which is *ca.* 98% larger (stronger) than the WBI for the non-bonding Os(1)⋯Os(3) atoms. The WBIs for the three Os–Sb and Os–P vectors are similar in magnitude and exhibit a mean index of 0.76 and 0.79, respectively. Finally, the mean index for the Os–C(benzyne) and the Os–H bonds is 0.71 and 0.39.

The spectroscopic data indicate that cluster 3 retains its solid-state structure in solution. The IR spectrum exhibits six *ν*(CO) bands within the range 2029–1919 cm^−1^, consistent with a product containing terminal CO ligands. The ^1^H NMR spectrum displays an upfield virtual triplet at −18.70 ppm (t, *J* 9.6 Hz) for the bridging hydride ligand, and the two multiplets at 2.28 and 2.05 ppm, each integrating to 1H, are attributed to the methylene protons of the dppm ligand. The remaining forty-nine hydrogens associated with the five aryl groups and benzyne moiety are found from 7.43 to 5.85 ppm. Finally, the pair of inequivalent phosphorus resonances appear as two doublets centered at 11.9 and 2.2 ppm (*J*_PP_ 55 Hz) in the ^31^P{^1^H} NMR spectrum.

### 3.2. Reaction of [HOs_3_(CO)_8_{μ_3_-Ph_2_PCH_2_PPh(C_6_H_4_-μ_2_,σ^1^)}] with SbPh_3_

The labile cluster [HOs_3_(CO)_8_{μ_3_-Ph_2_PCH_2_PPh(C_6_H_4_-μ_2_,σ^1^)}], whose ligand addition chemistry with CO and various phosphine donors is well-established,^26,38,39^ also reacts with two molar equivalents of SbPh_3_ at room temperature to afford [Os_3_(CO)_8_(SbPh_3_)(η^1^-Ph)(μ-SbPh_2_)(μ-dppm)] (4) in high yield (75%). When the reaction is repeated using a 1 : 1 ratio of starting cluster and SbPh_3_, cluster 4 was obtained in 45%. Cluster 4 has also been characterized by analytical and spectroscopic methods, and its molecular structure has been established by single-crystal X-ray diffraction analysis.

**Fig. 4:**
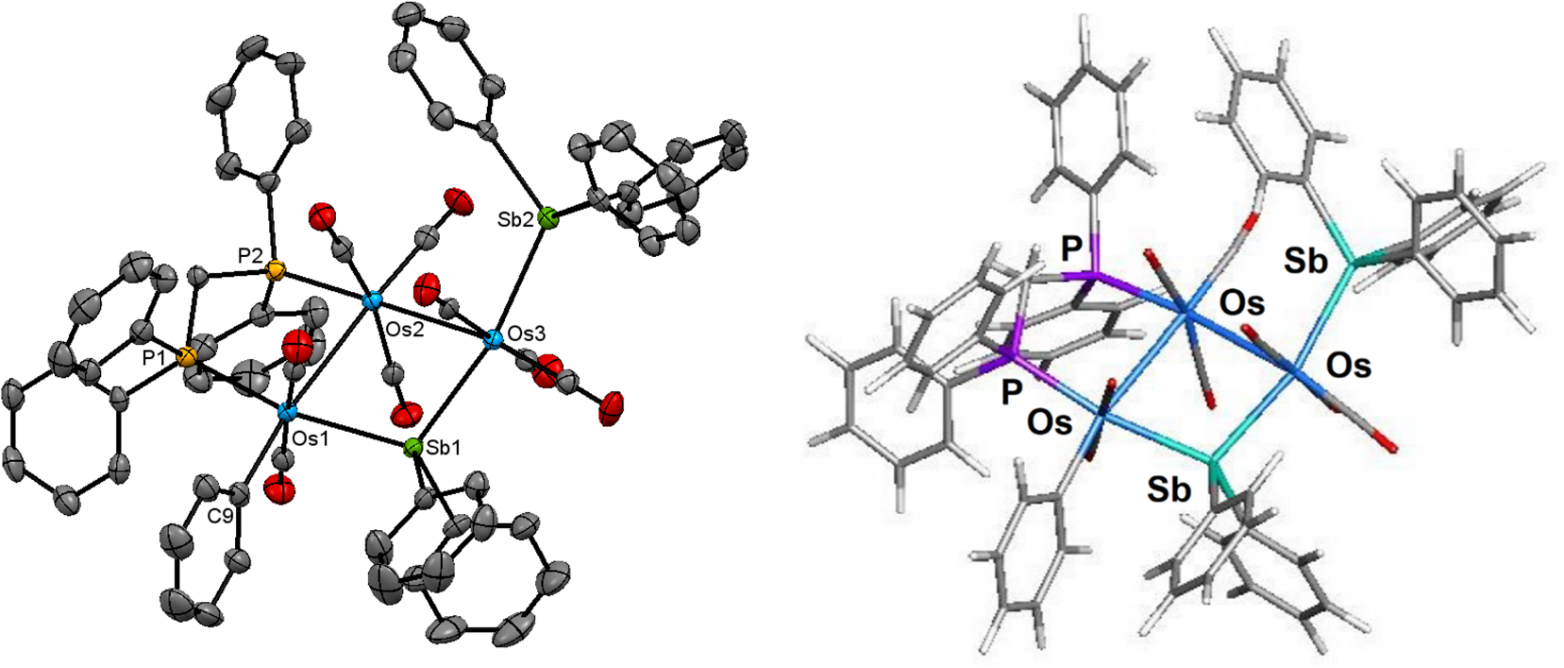
Molecular structure (left, 4) and M06-optimized structure (right, species D) of [Os_3_(CO)_8_(SbPh_3_) (η^1^-Ph)(μ-SbPh_2_)(μ-dppm)] showing 50% probability atomic displacement ellipsoids. Hydrogen atoms are omitted for clarity.

The molecular structure of 4 is depicted in Fig. 4. Cluster 4 contains 50 valence electrons, assuming the stibine and stibene ligands collectively contribute 5e to the total electron count. The molecule contains an open triosmium core with two almost equal osmium–osmium edges [Os(1)–Os(2) 3.0211(3) Å and Os(2)–Os(3) 3.0024(3) Å]. The dppm ligand bridges the Os(1)–Os(2) edge, while the non-bonding Os(1)⋯Os(3) edge is symmetrically bridged by the stibene ligand, exhibiting a mean Os–Sb(1) bond distance of 2.6587 Å. The distance between the non-bonding Os(1)⋯Os(3) atoms is 4.3501(5) Å which precludes any significant bonding interaction between these osmium atoms. The SbPh_3_ ligand is coordinated to Os(3) [Os(3)–Sb(2) 2.6468(4) Å] and is situated *trans* to the bridging SbPh_2_ ligand. The η^1^-phenyl ligand is coordinated to the Os(1) atom and resides in the equatorial plane defined by three osmium atoms. The DFT-optimized structure of species D is depicted alongside the solid-state structure in Fig. 4. The computed charges and WBIs parallel the data reported for species A–C. These data are included in Table 2.

**Scheme 1:**
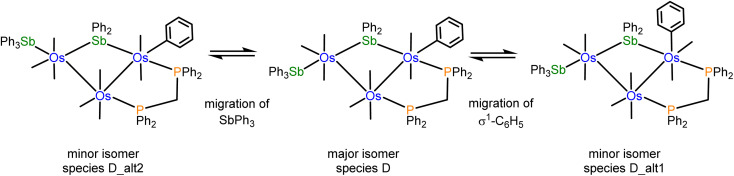
Different stereoisomers for [Os_3_(CO)_8_(SbPh_3_)(η^1^-Ph)(μ-SbPh_2_)(μ-dppm)] (4).

The NMR spectra of 4 indicate the presence of a pair of stereoisomers in solution. The ^31^P{^1^H} NMR spectrum displays two sets of resonances in a 10 : 1 ratio. The doublets at 13.8 and 0.2 ppm (*J*_PP_ 66 Hz) are attributed to the major isomer, while the doublets at 15.1 and 1.6 ppm (*J*_PP_ 66 Hz) are assigned to the minor isomer. Likewise, the aliphatic region of the ^1^H NMR spectrum shows two sets of resonances for the methylene protons of the dppm ligand. The virtual triplets at 4.01 ppm (*J* 10.4 Hz) and 3.94 ppm (*J* 10.4 Hz) are assigned to the major and minor isomers, respectively. Several possibilities may be proposed for the minor stereoisomer in 4. For example, the minor isomer may arise from a two-site exchange of an axial CO ligand and the σ^1^-Ph group at the Os(CO)_2_(SbPh_3_) moiety to yield a stereoisomer having an axial σ^1^-Ph ligand. Alternatively, a tripodal rotation involving two CO ligands and the SbPh_3_ donor at the Os(CO)_3_(SbPh_3_) moiety (Scheme 1) would afford a stereoisomer where the SbPh_3_ ligand is situated *cis* the bridging stibene ligand. Of the three stereoisomers in Scheme 1, the crystallographic structure is the most stable (species D) and is assigned as the major species in solution. The minor stereoisomer is attributed to the cluster containing an axial σ^1^-Ph ligand (D_alt1), lying 1.0 kcal mol^−1^ above species D. The other isomer (D_alt2) exhibits an equatorial SbPh_3_ ligand oriented *cis* to the bridging stibene ligand. D_alt2 lies 3.9 kcal mol^−1^ above D_alt1.

## 4. Discussion

**Scheme 2:**
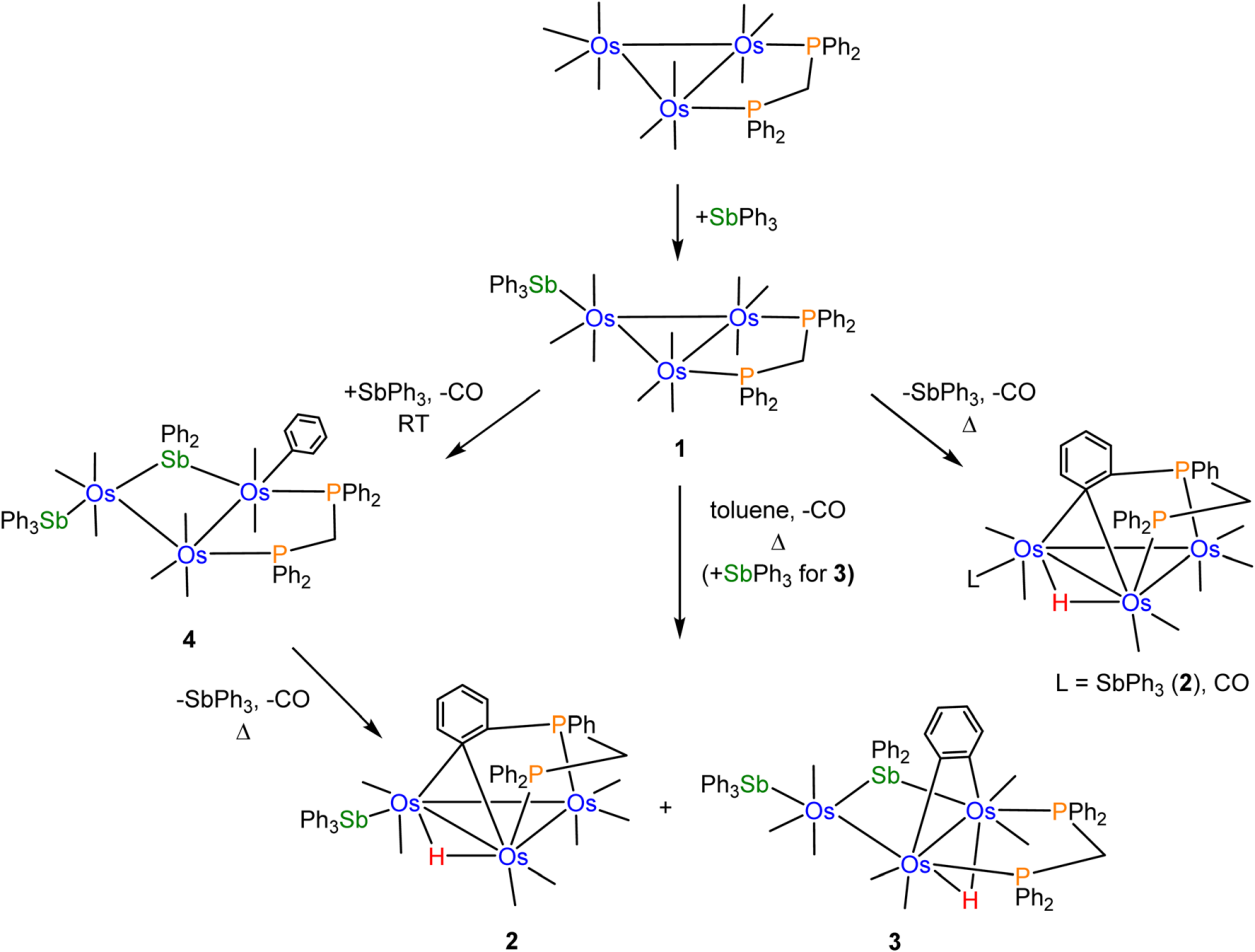
Pathways leading to clusters 1–4 staring from [Os_3_(CO)_10_(μ-dppm)] and SbPh_3_.

Scheme 2 shows the relationships between clusters 1–4 starting from [Os_3_(CO)_10_(μ-dppm)].

CO substitution in [Os_3_(CO)_10_(μ-dppm)] by SbPh_3_ gives cluster 1 using either thermal activation or oxidative decarbonylation using Me_3_NO. Control experiments using cluster 1 were conducted to confirm its relationship to clusters 2 and 3. Thermolysis of 1 in refluxing toluene afforded 2 as the major product (62%), together with a minor amount (22%) of the known benzylidene-bridged cluster [HOs_3_(CO)_8_{μ_3_-Ph_2_PCH_2_PPh(C_6_H_4_-μ_2_,σ^1^)}].^16^ These results confirm 1 as a precursor to 2 through loss of CO (2 equiv.), coupled with concomitant orthometalation of one of the phenyl rings of the dppm ligand. In the absence of added SbPh_3_, [HOs_3_(CO)_8_{μ_3_-Ph_2_PCH_2_PPh(C_6_H_4_-μ_2_,σ^1^)}] forms as a minor product from the competitive loss of SbPh_3_*versus* CO in 1, followed by orthometalation of one of the aryl rings on the dppm ligand. [HOs_3_(CO)_8_{μ_3_-Ph_2_PCH_2_PPh(C_6_H_4_-μ_2_,σ^1^)}] was not observed when [Os_3_(CO)_10_(μ-dppm)] was treated with SbPh_3_ in refluxing toluene. The competitive pathway involving the loss of SbPh_3_ in the thermolysis of 1 is efficiently suppressed in the presence of SbPh_3_ (2 equiv.). In a separate experiment that was monitored by TLC, we confirmed that the reaction between 1 and SbPh_3_ at 110 °C also furnished clusters 2 (14%) and 3 (20%) without a trace of [HOs_3_(CO)_8_{μ_3_-Ph_2_PCH_2_PPh(C_6_H_4_-μ_2_,σ^1^)}].

Control experiments confirmed that cluster 4 is converted into 2 (63%) and 3 (30%) in moderate yield in refluxing toluene. The η^1^-C_6_H_5_ ligand in 4 undergoes reductive elimination with the bridging stibene ligand, followed by metalation of an aryl ring on the dppm ligand and release of one SbPh_3_ ligand to ultimately give 2. Competitive with this route is the loss of one CO from 4, followed by orthometalation of the η^1^-C_6_H_5_ ligand, to give 3. The formation of 4 from [Os_3_(CO)_10_(μ-dppm)] and SbPh_3_ (excess) at 110 °C is short-lived, and it transforms into clusters 2 and 3. Independent experiments on purified samples of 2 and 3 in toluene (110 °C) also afforded 4 with significant material loss noted. These results are understood within a kinetic framework where the rates of decomposition of clusters 2 and 3 are faster than cluster 4.

Facile Sb–Ph bond cleavage was observed in the control reaction involving 1 and SbPh_3_ at room temperature. Here cluster 4 is produced in nearly quantitative yield. We have evaluated the thermodynamics for the conversion of 4 → 2 (plus CO and SbPh_3_) and 3 (plus CO), and electronic structure calculations confirmed 4 as thermodynamically more stable. The formation of 2 along with the liberated CO and SbPh_3_ ligands from 4 is endergonic by 28.3 kcal mol^−1^, while the formation of 3 and CO lies 33.1 kcal mol^−1^ above species D (cluster 4). The formation of these products is driven, in part, by entropic contributions involving the release of CO.

**Scheme 3:**
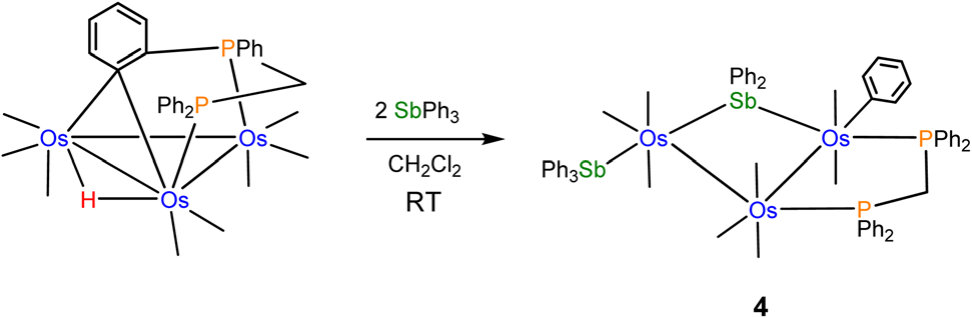
Formation of 4 from [HOs_3_(CO)_8_{μ_3_-Ph_2_PCH_2_PPh(C_6_H_4_-μ_2_,σ^1^)}] and SbPh_3_.

The reactivity of the labile cluster [HOs_3_(CO)_8_{μ_3_-Ph_2_PCH_2_PPh(C_6_H_4_-μ_2_,σ^1^)}] with SbPh_3_ was also investigated. The reaction proceeds rapidly at room temperature to give cluster 4 as the major product. Scheme 3 illustrates this reaction. Reductive coupling of the hydride and benzylidene moieties regenerates the dppm ligand, followed by stibine coordination and oxidative Sb–Ph bond cleavage.

## 5. Conclusions

Four new triosmium clusters containing Sb-donor ligands, namely [Os_3_(CO)_9_(SbPh_3_)(μ-dppm)] (1), [HOs_3_(CO)_7_(SbPh_3_){μ_3_-Ph_2_PCH_2_PPh(C_6_H_4_-μ_2_,σ^1^)}] (2), [HOs_3_(CO)_7_(SbPh_3_)(μ-C_6_H_4_)(μ-SbPh_2_)(μ-dppm)] (3) and [Os_3_(CO)_8_(η^1^-Ph)(SbPh_3_)(μ-SbPh_2_)(μ-dppm)] (4) have been isolated and characterized from the reactions of SbPh_3_ with [Os_3_(CO)_10_(μ-dppm)] and [HOs_3_(CO)_8_{μ_3_-Ph_2_PCH_2_PPh(C_6_H_4_-μ_2_,σ^1^)}]. The molecular structure of each new cluster has been determined by single-crystal X-ray diffraction analysis. The reaction of [Os_3_(CO)_10_(μ-dppm)] with SbPh_3_ at 110 °C gives 1–3, with the simple substitution product 1 acting as a direct precursor to clusters 2 and 3. Cluster 3 represents a rare example of triosmium complexes containing a bridging C_6_H_4_ (benzyne) group with ancillary stibine and stibene ligands. In contrast, the reaction between [HOs_3_(CO)_8_{μ_3_-Ph_2_PCH_2_PPh(C_6_H_4_-μ_2_,σ^1^)}] and SbPh_3_ occurs at room temperature and furnishes 4, which contains a η^1^-C_6_H_5_ ligand generated *via* Sb–Ph bond cleavage of one SbPh_3_ ligand. Control experiments revealed that 4 is converted to clusters 2 and 3 in refluxing toluene. Kinetic studies on the reactions reported here are planned for the future. These results will be published in due course.

## Conflicts of interest

The authors declare that they have no known competing financial interests or personal relationships that could have appeared to influence the work reported in this paper.

## Supplementary Material

RA-013-D2RA07284J-s001

RA-013-D2RA07284J-s002
