# Supplementary material for: Reactions of diphosphine-stabilized Os3 clusters with triphenylantimony: syntheses and structures of new antimony-containing Os3 clusters via Sb–Ph bond cleavage
Source: RSC Adv. 2023 Jan 18;13(5):2841–51. doi: 10.1039/d2ra07284j (PMC9845985; doi:10.1039/d2ra07284j)
Supplement: RA-013-D2RA07284J-s001 [file RA-013-D2RA07284J-s001.pdf]

Electronic Supplementary Material (ESI) for RSC Advances.
This journal is © The Royal Society of Chemistry 2023

Supplementary Information (SI)

Reactions of diphosphine-stabilized Os_3 clusters with triphenylantimony: Syntheses and structures of new antimony-containing Os_3 clusters via Sb-Ph bond cleavage

Fahmida Islam [a], Md. Sohag Hasan [a], Shishir Ghosh [a,*], Michael G. Richmond [b], Shariff E. Kabir [a,c,*], Herbert W. Roesky [c,*]

[a] *Department of Chemistry, Jahangirnagar University, Savar, Dhaka 1342, Bangladesh*
[b] *Department of Chemistry, University of North Texas, 1155 Union Circle, Box 305070, Denton, TX 76203, USA*
[c] *Institute of Inorganic Chemistry, Georg-August University of Göttingen, Tammannnstr 4, 37077 Göttingen, Germany*

*Corresponding authors.
E-mail addresses: sghosh_006@yahoo.com (S. Ghosh); skabir_ju@yahoo.com (S.E. Kabir); hroesky@gwdg.de (H.W. Roesky)

List of Figures and Tables

Fig. S1. Aliphatic region of the ^{1}H NMR spectrum of $[Os_3(CO)_9(SbPh_3)(\mu\text{-dppm})]$ (**1**) in $CDCl_3$.

Fig. S2. Aromatic region of the ^{1}H NMR spectrum of $[Os_3(CO)_9(SbPh_3)(\mu\text{-dppm})]$ (**1**) in $CDCl_3$.

Fig. S3. $^{31}P\{^1H\}$ NMR spectrum of $[Os_3(CO)_9(SbPh_3)(\mu\text{-dppm})]$ (**1**) in $CDCl_3$.

Fig. S4. Hydride region of the ^{1}H NMR spectrum of $[HOs_3(CO)_7(SbPh_3)\{\mu_3\text{-}Ph_2PCH_2(Ph)C_6H_4\}]$ (**2**) in CD_2Cl_2.

Fig. S5. Aliphatic region of the ^{1}H NMR spectrum of $[HOs_3(CO)_7(SbPh_3)\{\mu_3\text{-}Ph_2PCH_2(Ph)C_6H_4\}]$ (**2**) in CD_2Cl_2.

Fig. S6. Aromatic region of the ^{1}H NMR spectrum of $[HOs_3(CO)_7(SbPh_3)\{\mu_3\text{-}Ph_2PCH_2(Ph)C_6H_4\}]$ (**2**) in CD_2Cl_2.

Fig. S7. $^{31}P\{^1H\}$ NMR spectrum of $[HOs_3(CO)_7(SbPh_3)\{\mu_3\text{-}Ph_2PCH_2(Ph)C_6H_4\}]$ (**2**) in CD_2Cl_2.

Fig. S8. Hydride region of the ^{1}H NMR spectrum of $[HOs_3(CO)_7(SbPh_3)(\mu,\eta^2\text{-}C_6H_4)(\mu\text{-}SbPh_2)(\mu\text{-dppm})]$ (**3**) in $CDCl_3$.

Fig. S9. Aliphatic region of the ^{1}H NMR spectrum of $[HOs_3(CO)_7(SbPh_3)(\mu,\eta^2\text{-}C_6H_4)(\mu\text{-}SbPh_2)(\mu\text{-dppm})]$ (**3**) in $CDCl_3$.

Fig. S10. Aromatic region of the ^{1}H NMR spectrum of $[HOs_3(CO)_7(SbPh_3)(\mu,\eta^2\text{-}C_6H_4)(\mu\text{-}SbPh_2)(\mu\text{-dppm})]$ (**3**) in $CDCl_3$.

Fig. S11. $^{31}P\{^1H\}$ NMR spectrum of $[HOs_3(CO)_7(SbPh_3)(\mu,\eta^2\text{-}C_6H_4)(\mu\text{-}SbPh_2)(\mu\text{-dppm})]$ (**3**) in $CDCl_3$.

Fig. S12. Aliphatic region of the ^{1}H NMR spectrum of $[Os_3(CO)_8(\eta^1\text{-}Ph)(SbPh_3)(\mu\text{-}SbPh_2)(\mu\text{-dppm})]$ (**4**) in CD_2Cl_2.

Fig. S13. Aromatic region of the ^{1}H NMR spectrum of $[Os_3(CO)_8(\eta^1\text{-}Ph)(SbPh_3)(\mu\text{-}SbPh_2)(\mu\text{-dppm})]$ (**4**) in CD_2Cl_2.

Fig. S14. $^{31}P\{^1H\}$ NMR spectrum of $[Os_3(CO)_8(\eta^1\text{-}Ph)(SbPh_3)(\mu\text{-}SbPh_2)(\mu\text{-dppm})]$ (**4**) in CD_2Cl_2.

Table S1. Selected bond distances (Å) and angles (°) for clusters **1**-**4**.

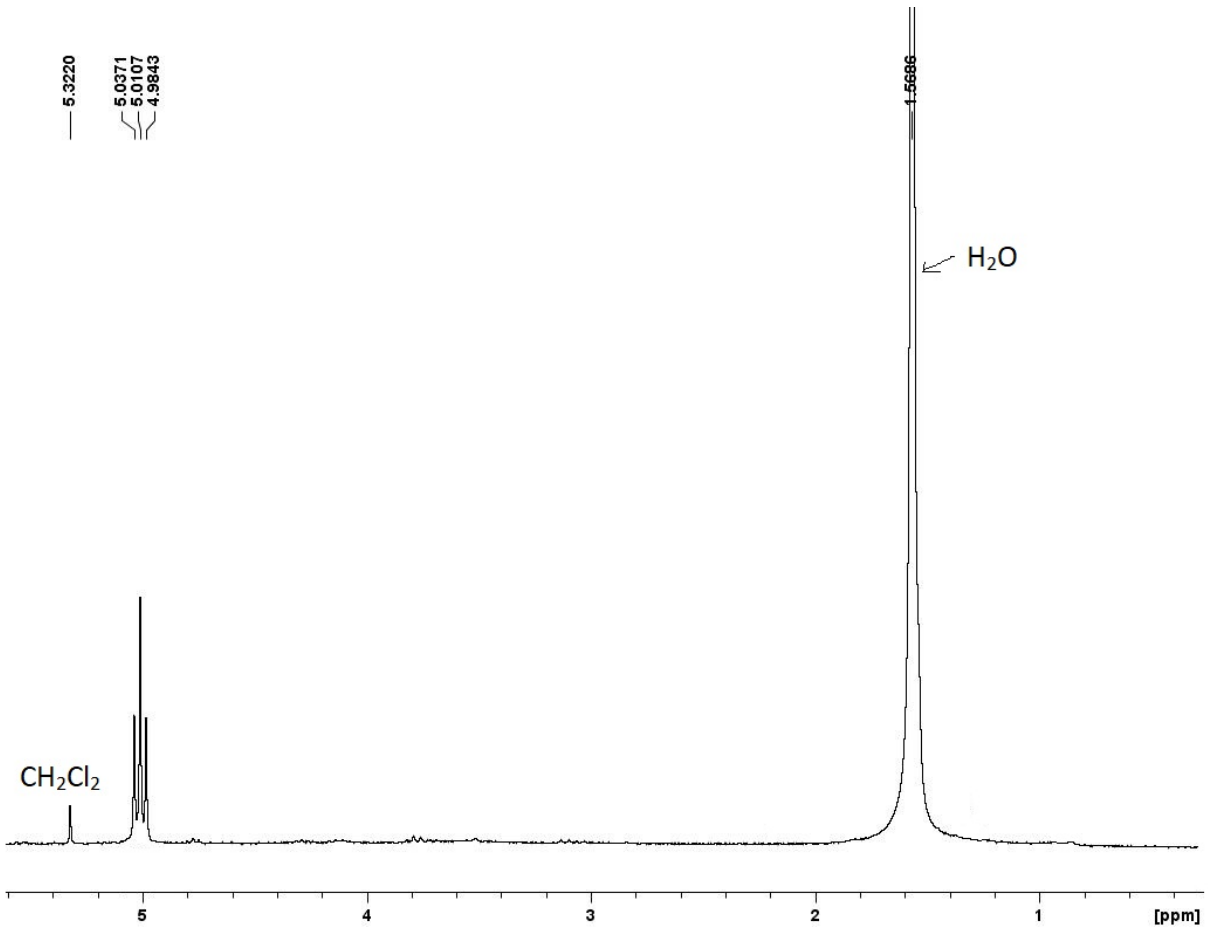

Fig. S1. Aliphatic region of the ^{1}H NMR spectrum of [$Os_3(CO)_9(SbPh_3)(\mu$-dppm)] (**1**) in $CDCl_3$.

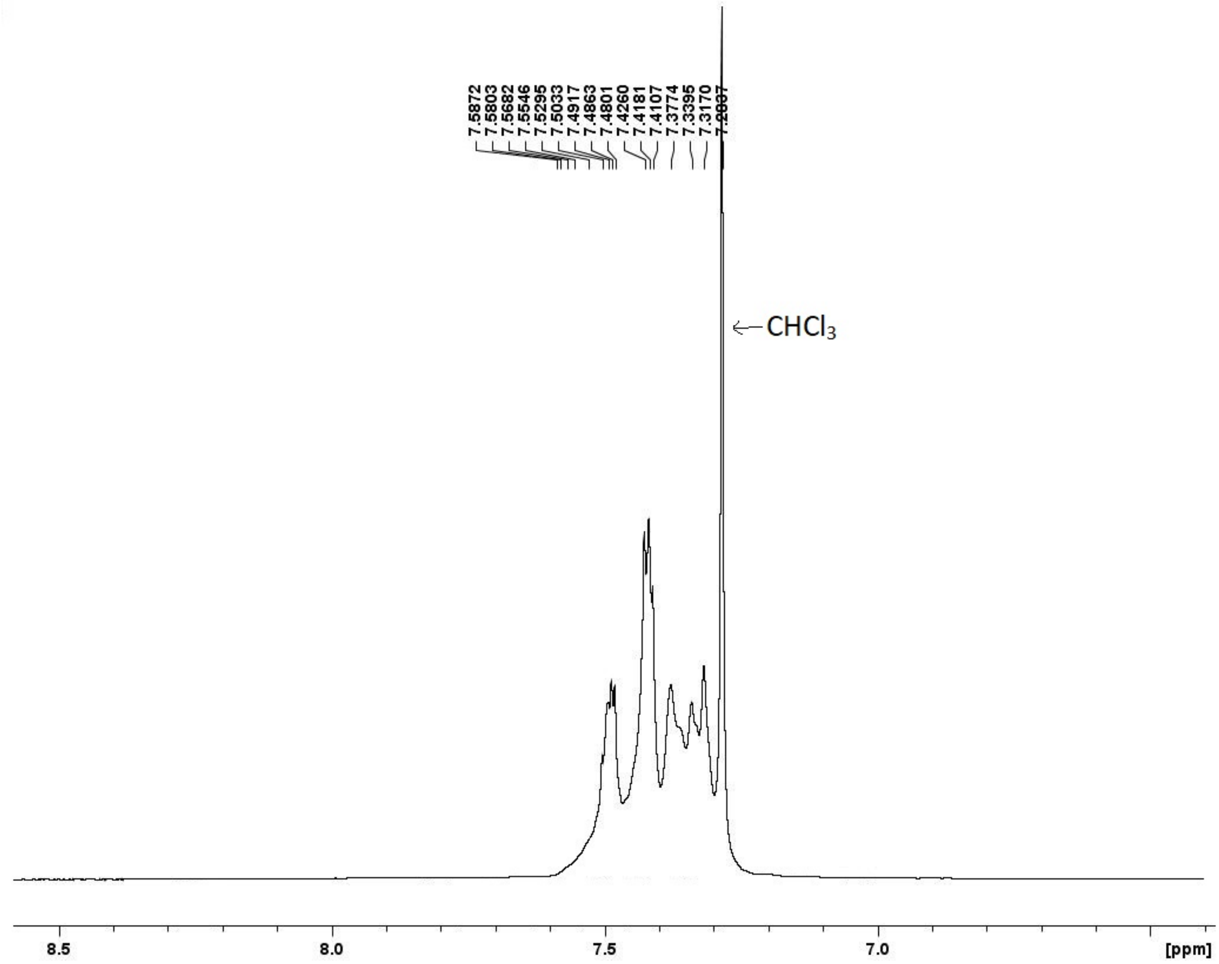

Fig. S2. Aromatic region of the ^{1}H NMR spectrum of $[Os_3(CO)_9(SbPh_3)(\mu\text{-dppm})]$ (**1**) in $CDCl_3$.

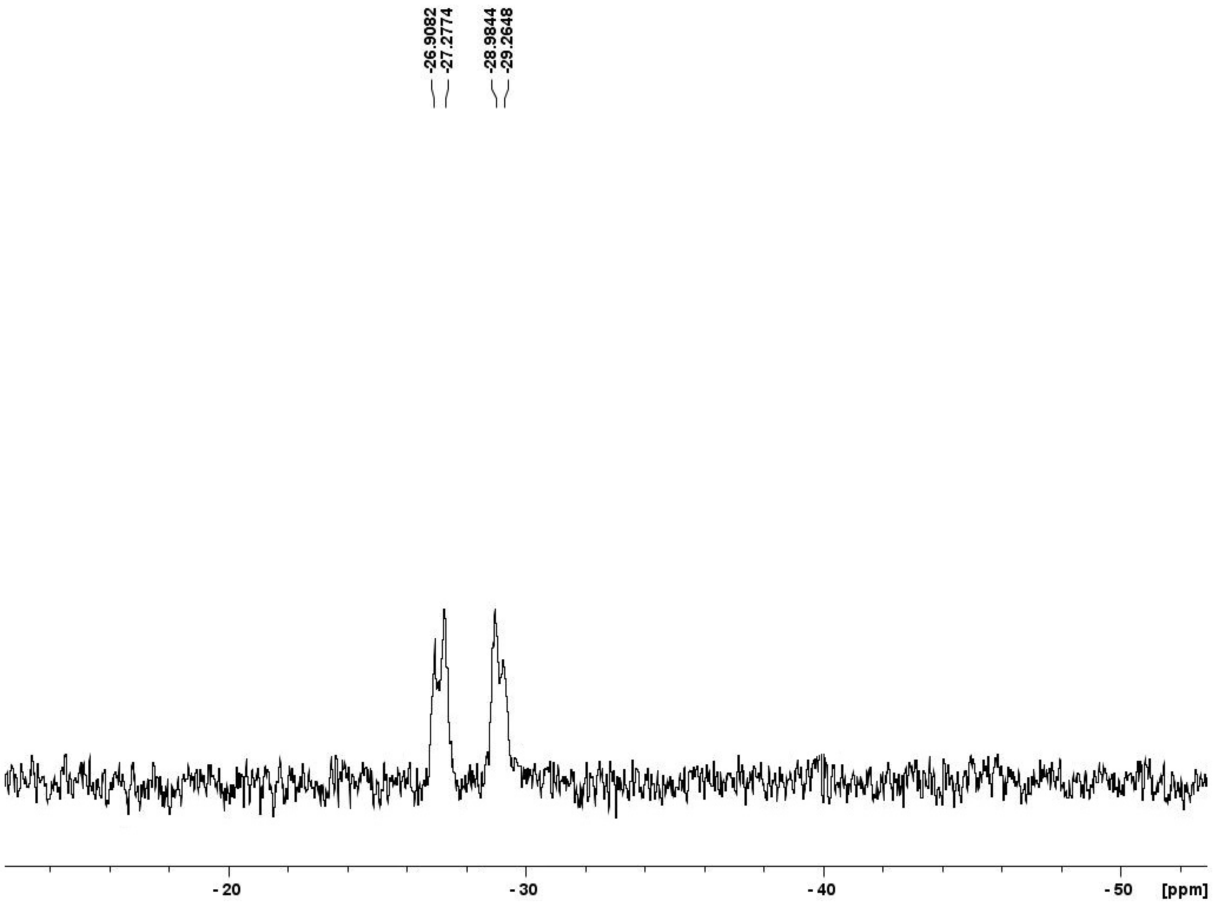

Fig. S3. $^{31}P\{^{1}H\}$ NMR spectrum of $[Os_3(CO)_9(SbPh_3)(\mu\text{-dppm})]$ (**1**) in $CDCl_3$.

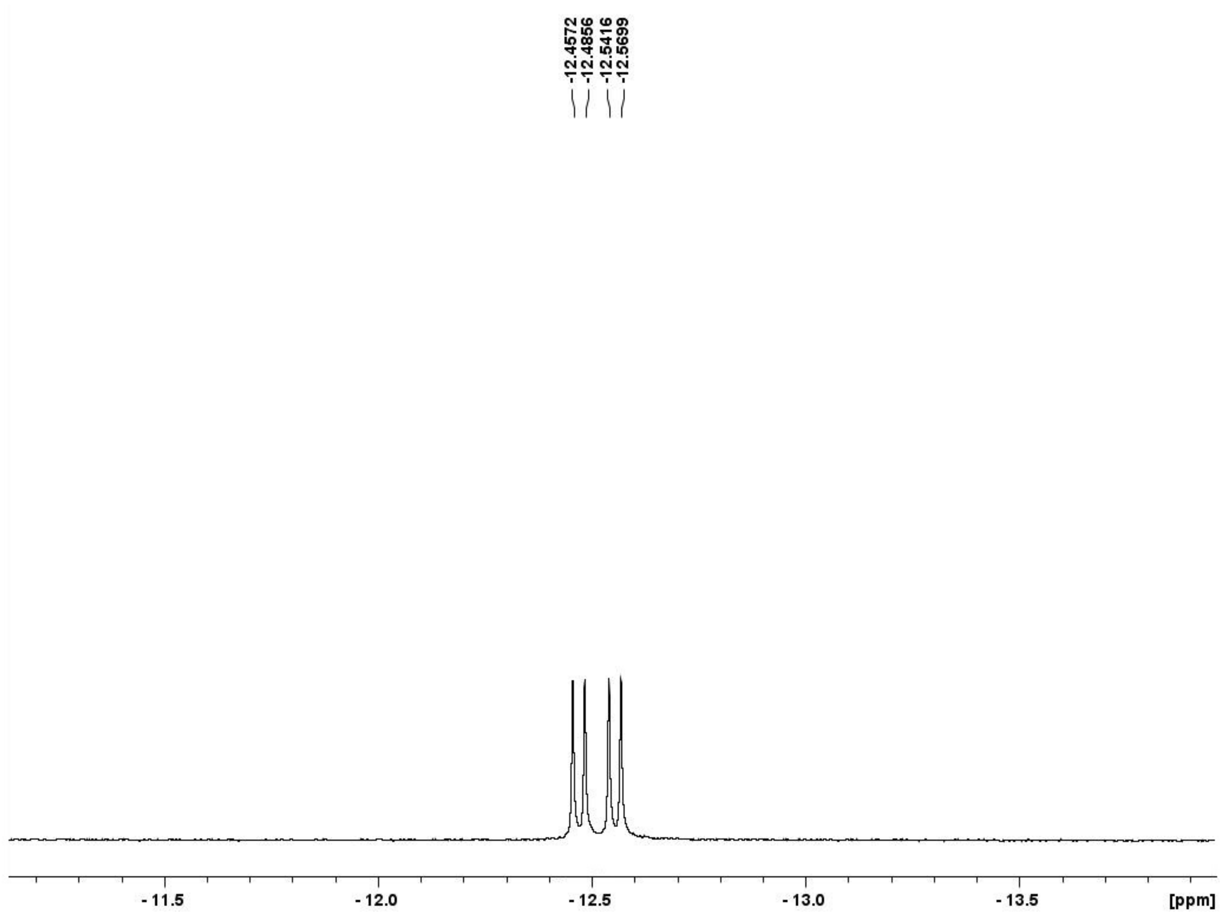

Fig. S4. Hydride region of the ^{1}H NMR spectrum of [$HOs_3(CO)_7(SbPh_3)$\{μ_3-$Ph_2PCH_2(Ph)C_6H_4$\}] (**2**) in CD_2Cl_2.

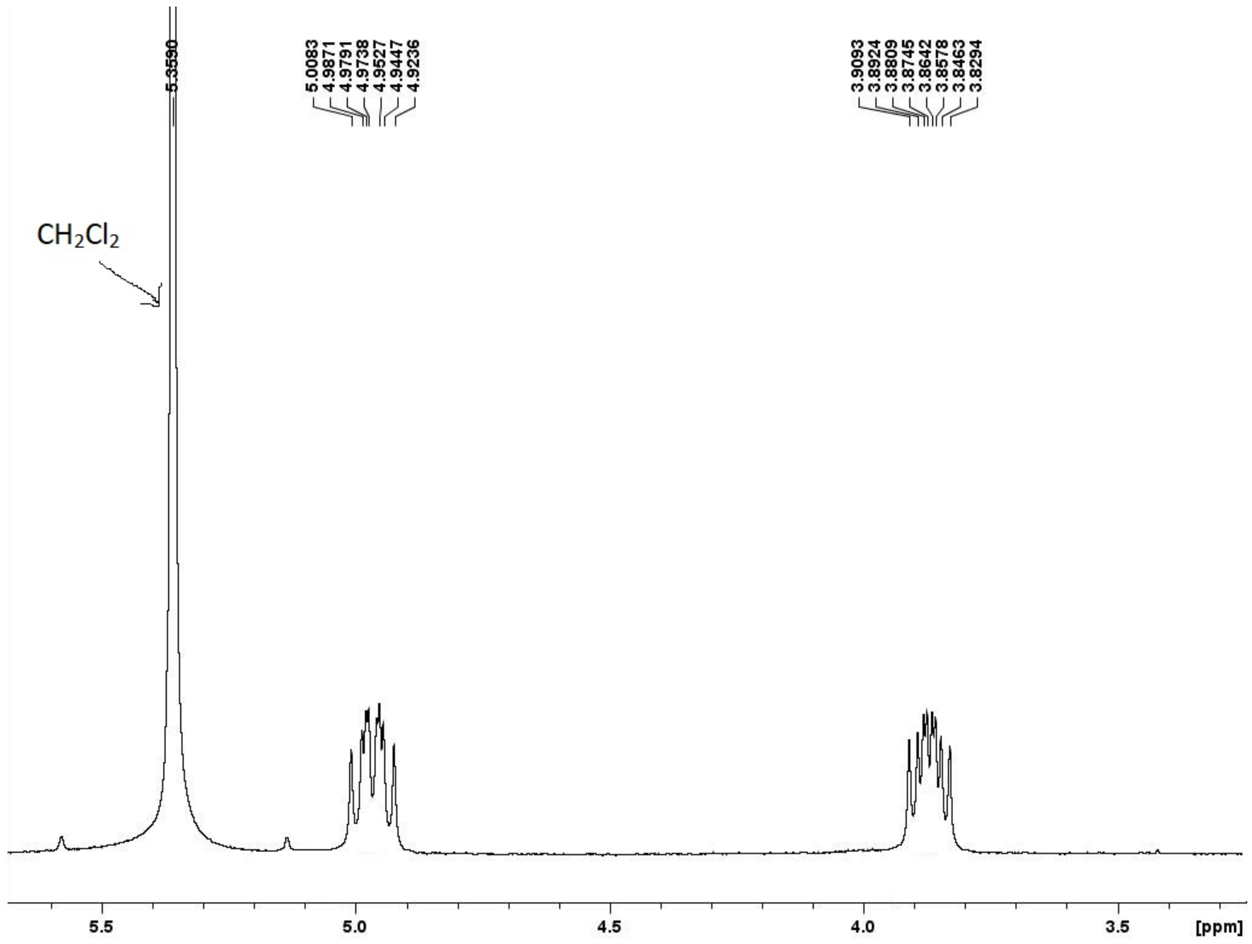

Fig. S5. Aliphatic region of the ^{1}H NMR spectrum of [$HOs_3(CO)_7(SbPh_3)$\{μ_3-$Ph_2PCH_2(Ph)C_6H_4$\}] (**2**) in CD_2Cl_2.

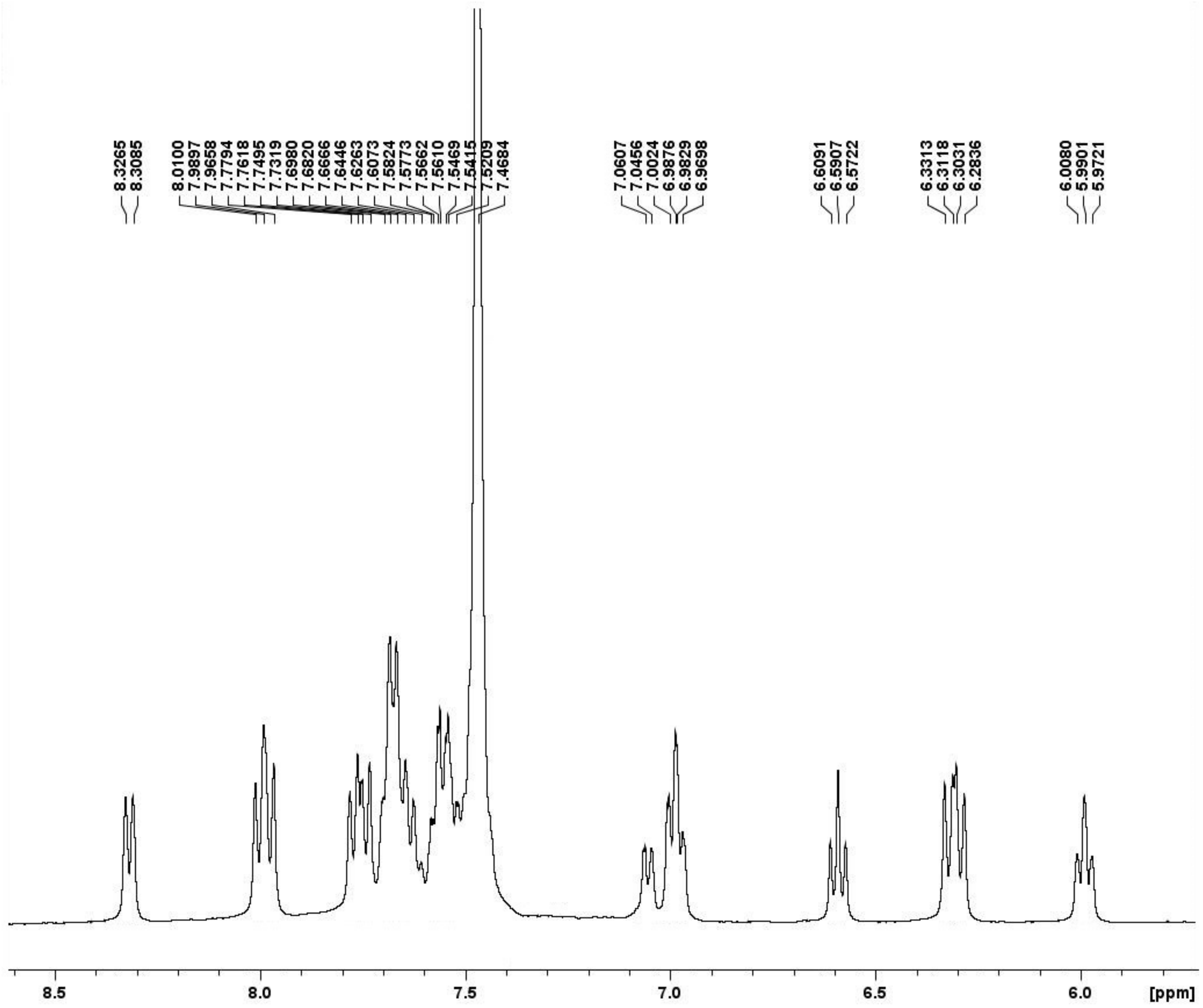

Fig. S6. Aromatic region of the ^{1}H NMR spectrum of $[HOs_3(CO)_7(SbPh_3)\{\mu_3\text{-}Ph_2PCH_2(Ph)C_6H_4\}]$ (**2**) in CD_2Cl_2.

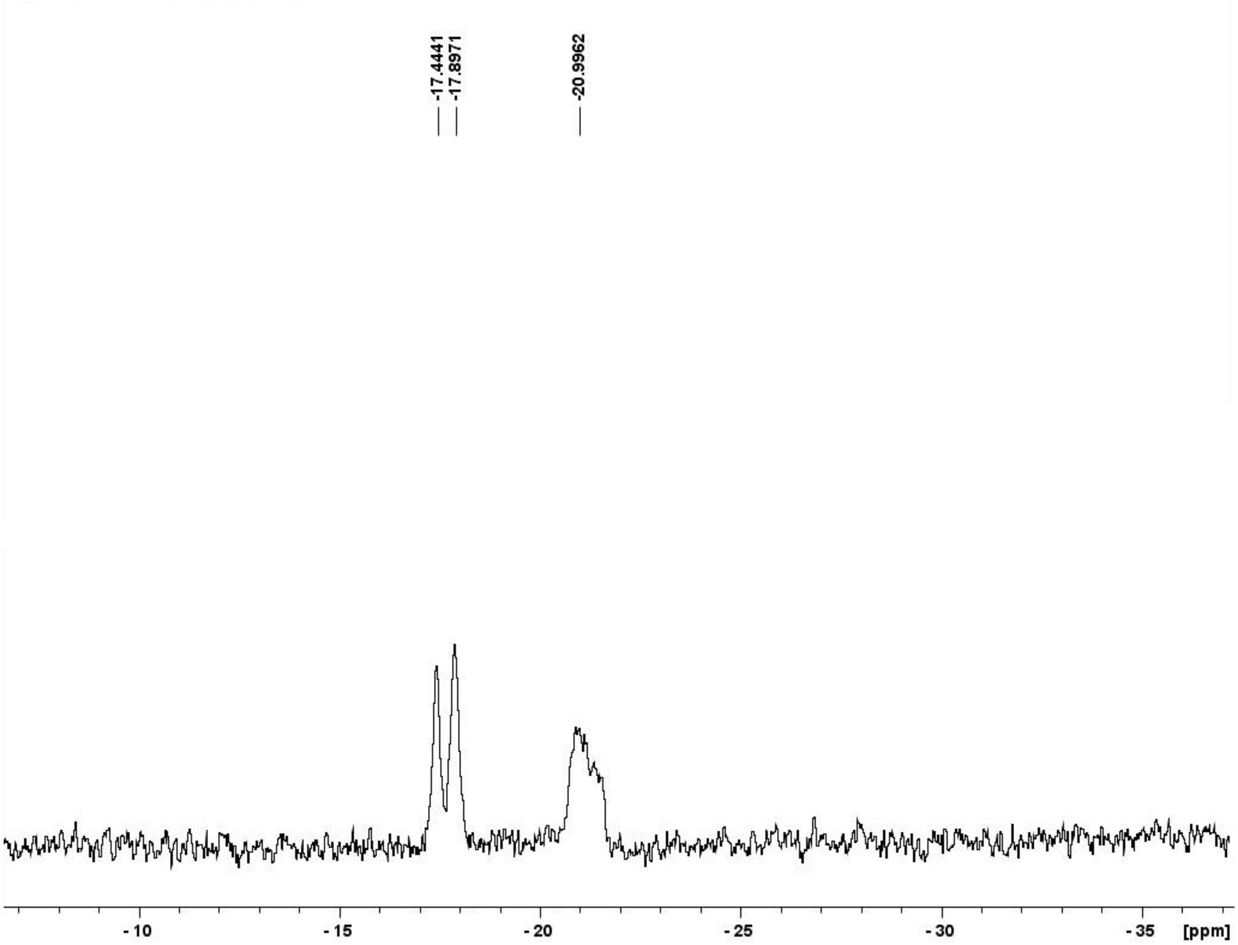

Fig. S7. $^{31}P\{^1H\}$ NMR spectrum of $[HOs_3(CO)_7(SbPh_3)\{\mu_3\text{-}Ph_2PCH_2(Ph)C_6H_4\}]$ (**2**) in CD_2Cl_2.

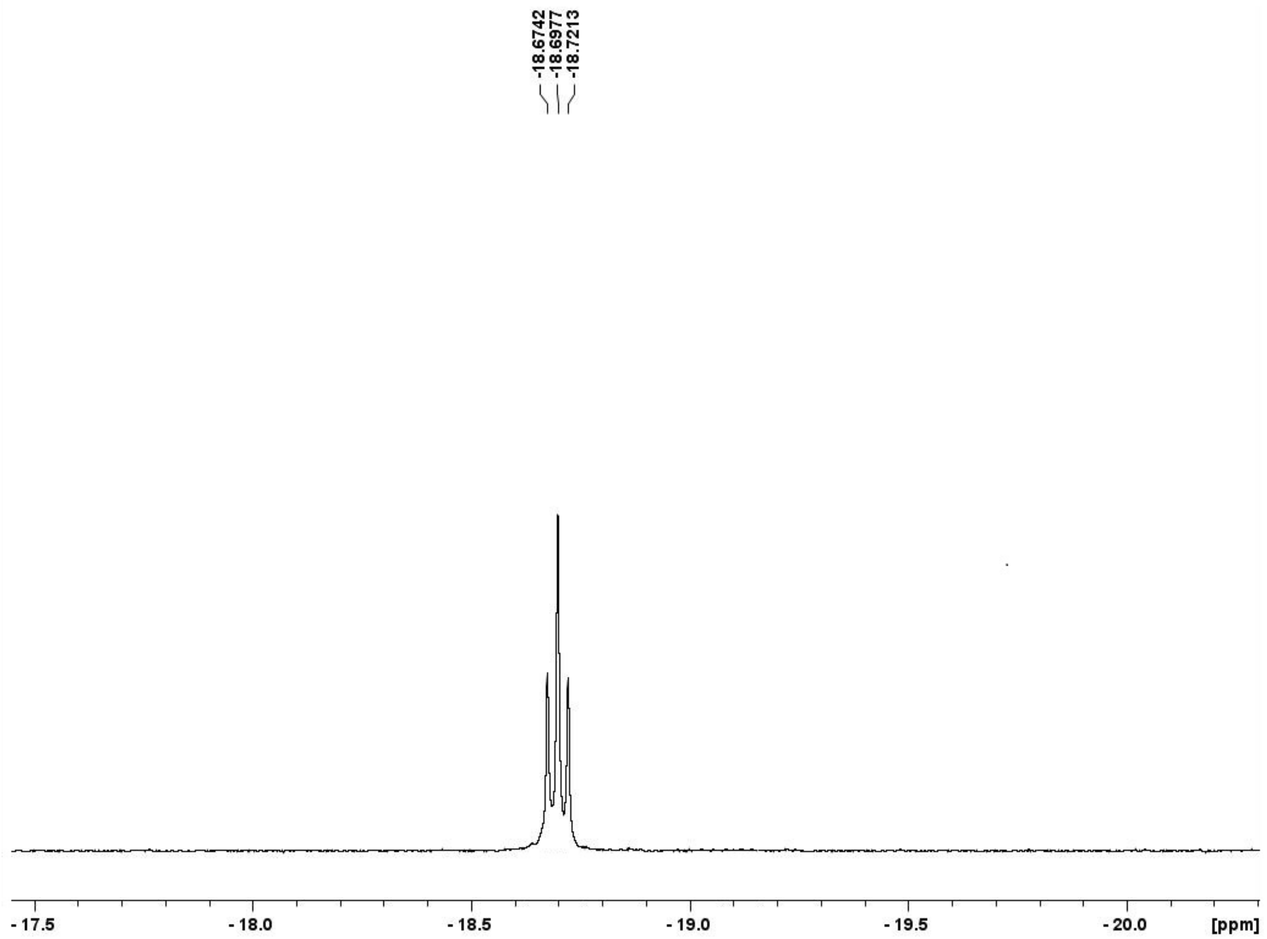

Fig. S8. Hydride region of the ^{1}H NMR spectrum of $[HOs_3(CO)_7(SbPh_3)(\mu,\eta^2\text{-}C_6H_4)(\mu\text{-}SbPh_2)(\mu\text{-dppm})]$ (**3**) in $CDCl_3$.

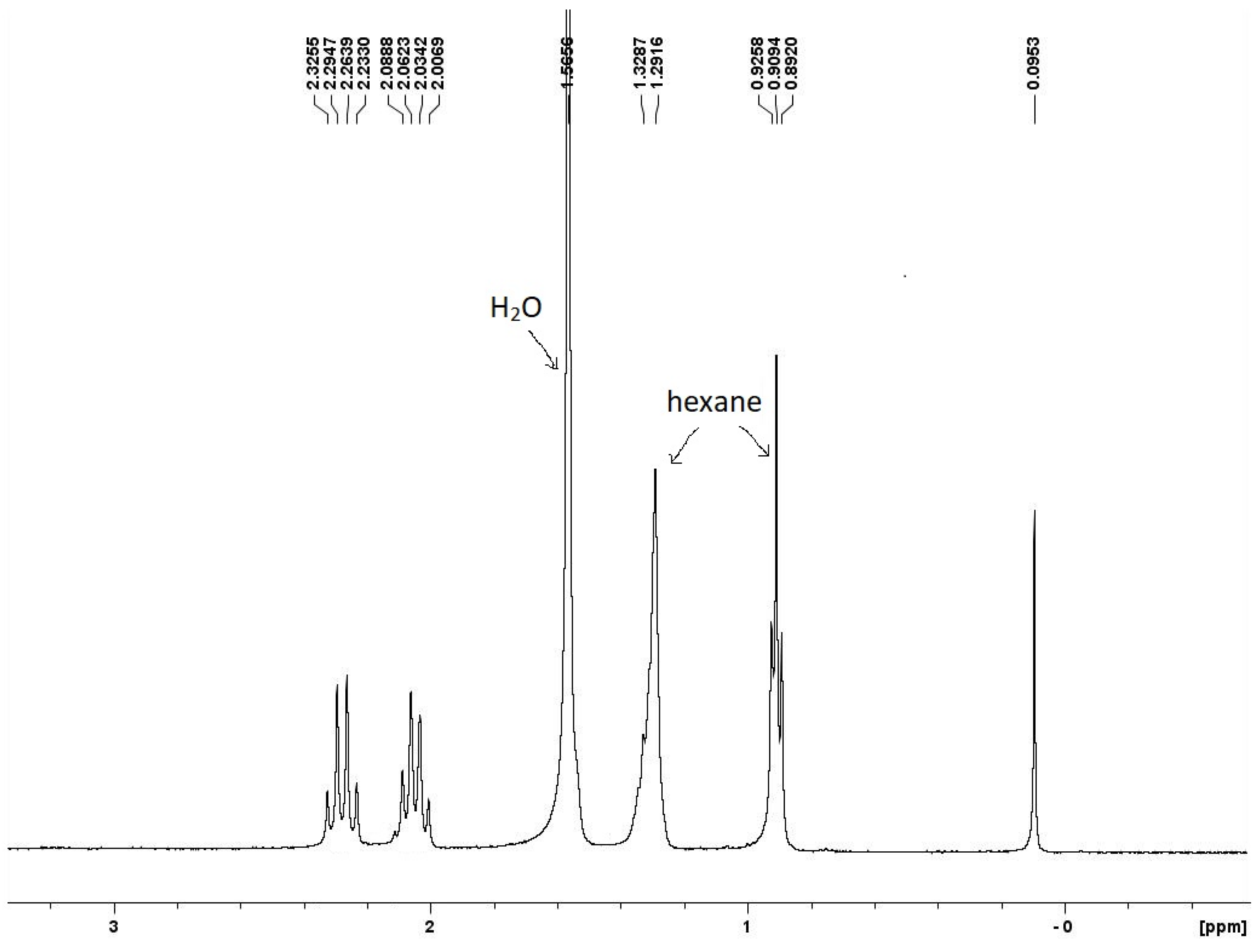

Fig. S9. Aliphatic region of the ^{1}H NMR spectrum of $[HOs_3(CO)_7(SbPh_3)(\mu,\eta^2\text{-}C_6H_4)(\mu\text{-}SbPh_2)(\mu\text{-dppm})]$ (**3**) in $CDCl_3$.

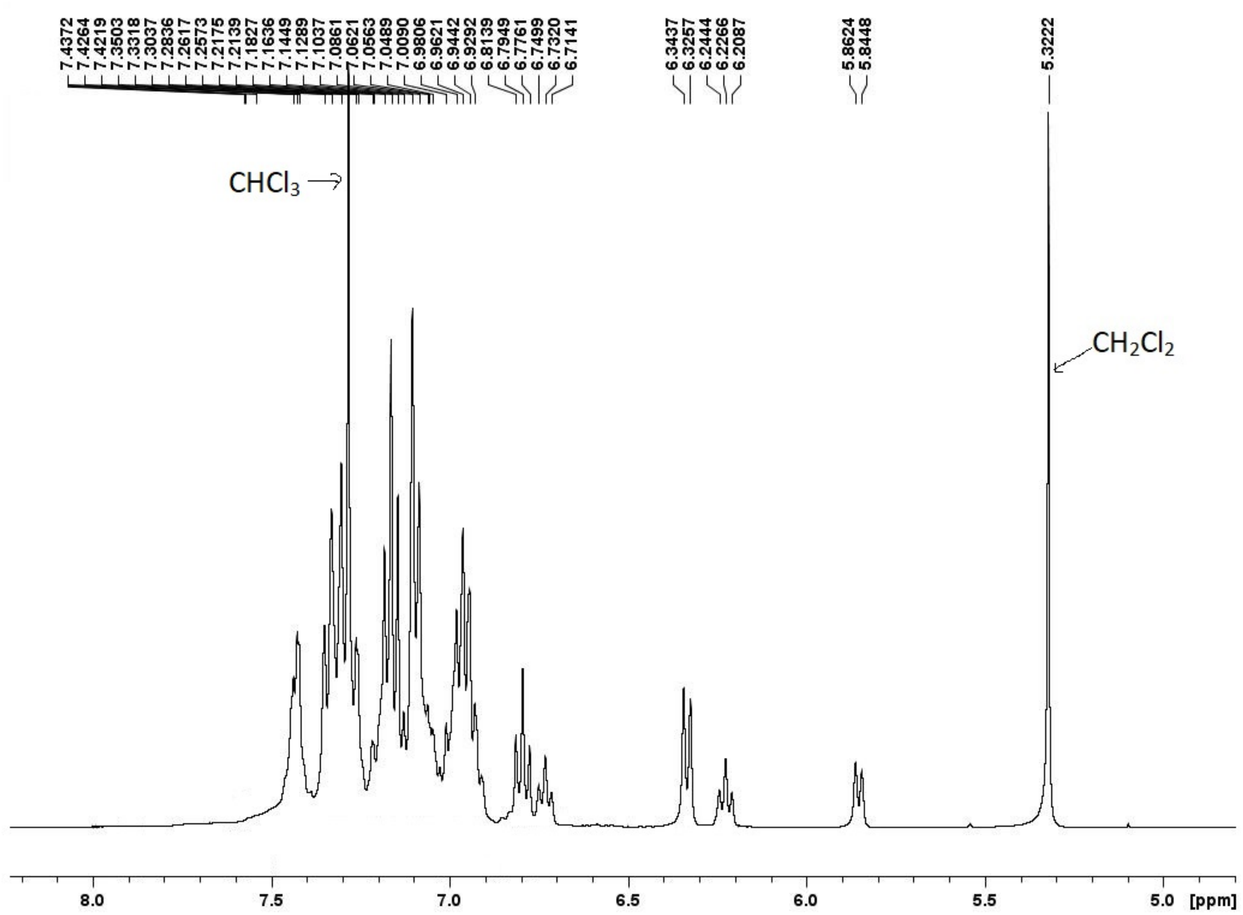

Fig. S10. Aromatic region of the ^{1}H NMR spectrum of [$HOs_3(CO)_7(SbPh_3)(\mu,\eta^2$-$C_6H_4)(\mu$-$SbPh_2)(\mu$-dppm)] (**3**) in $CDCl_3$.

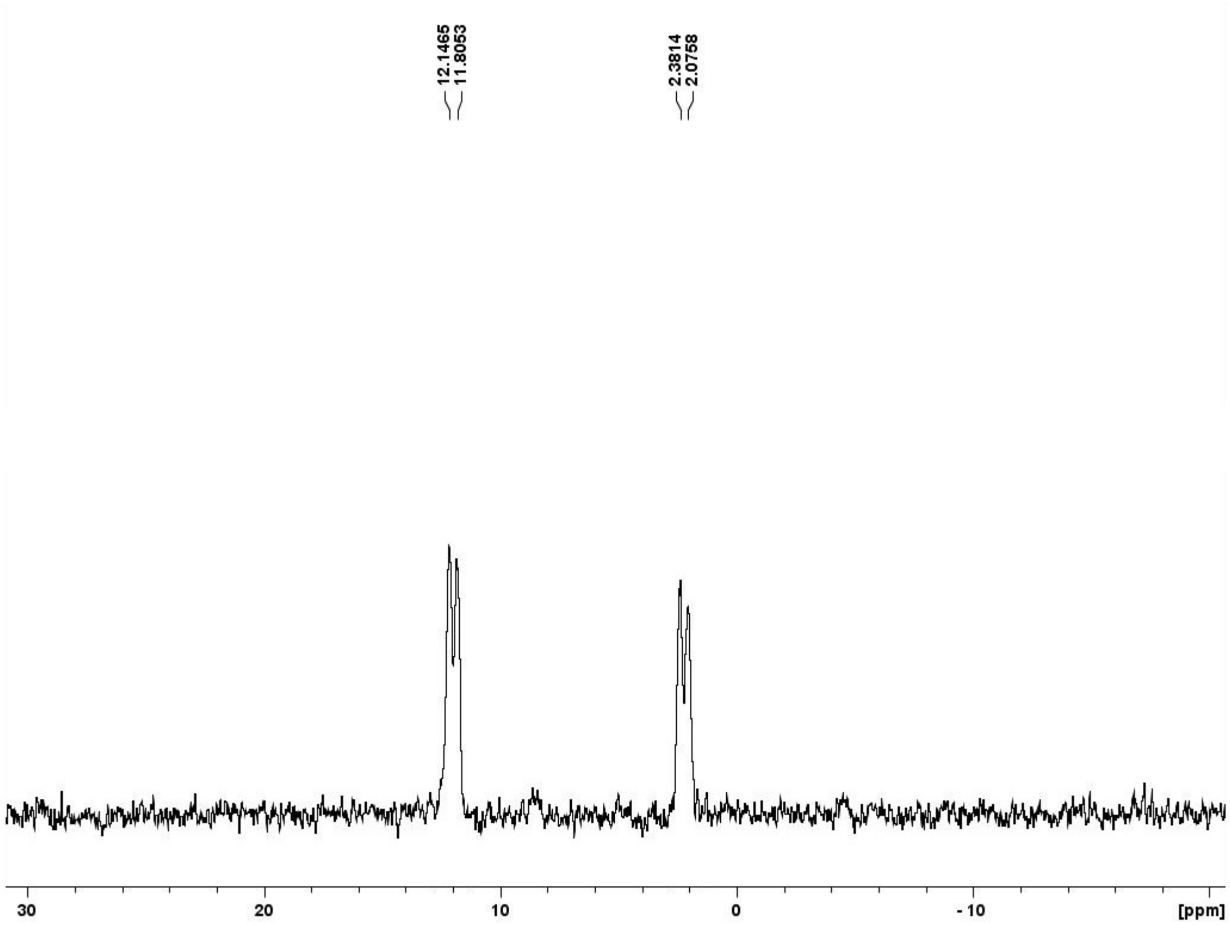

Fig. S11. $^{31}P\{^1H\}$ NMR spectrum of $[HOs_3(CO)_7(SbPh_3)(\mu,\eta^2\text{-}C_6H_4)(\mu\text{-}SbPh_2)(\mu\text{-dppm})]$ (**3**) in $CDCl_3$.

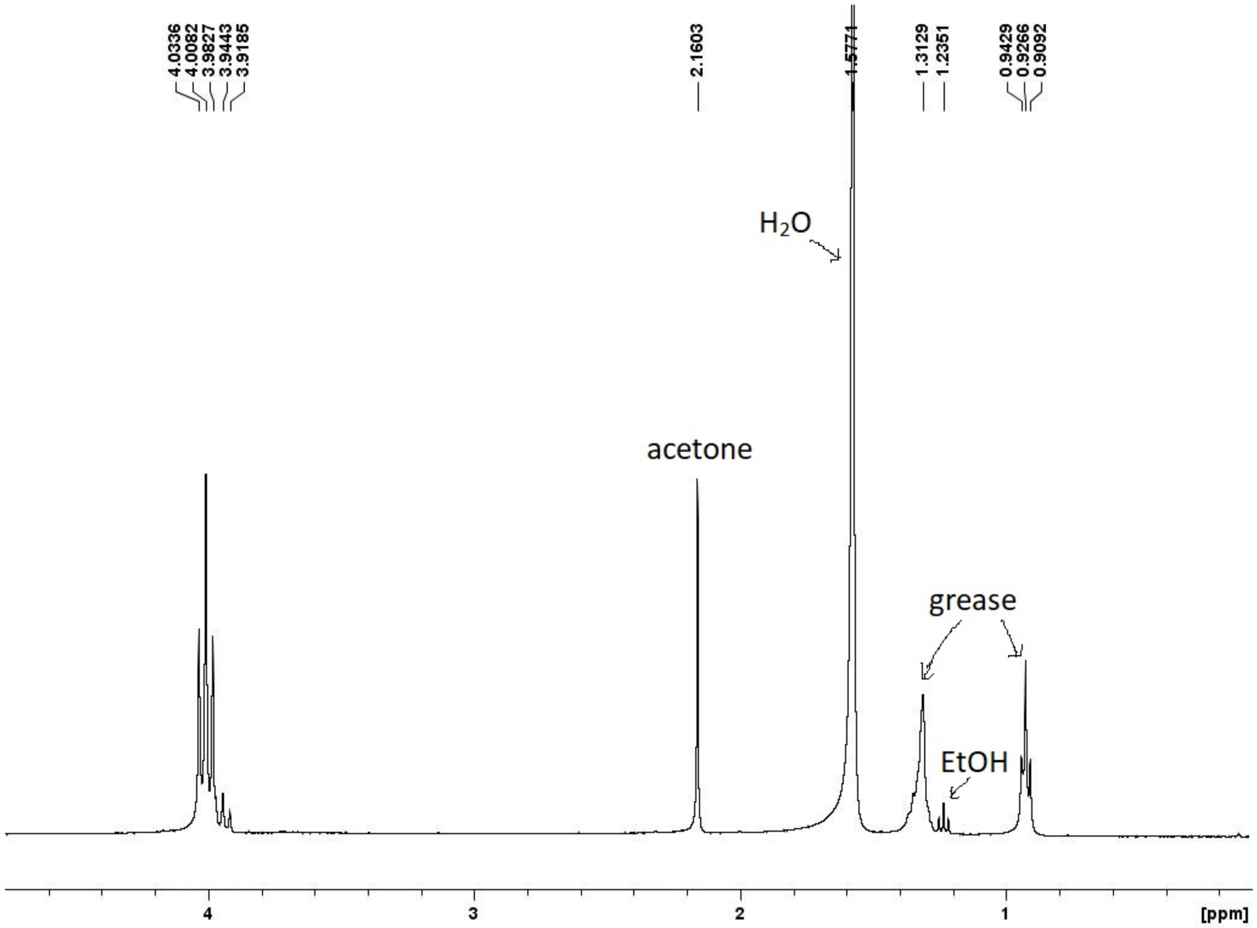

Fig. S12. Aliphatic region of the ^{1}H NMR spectrum of [$Os_3(CO)_8(\eta^1$-Ph)($SbPh_3$)(μ-$SbPh_2$)(μ-dppm)] (**4**) in CD_2Cl_2.

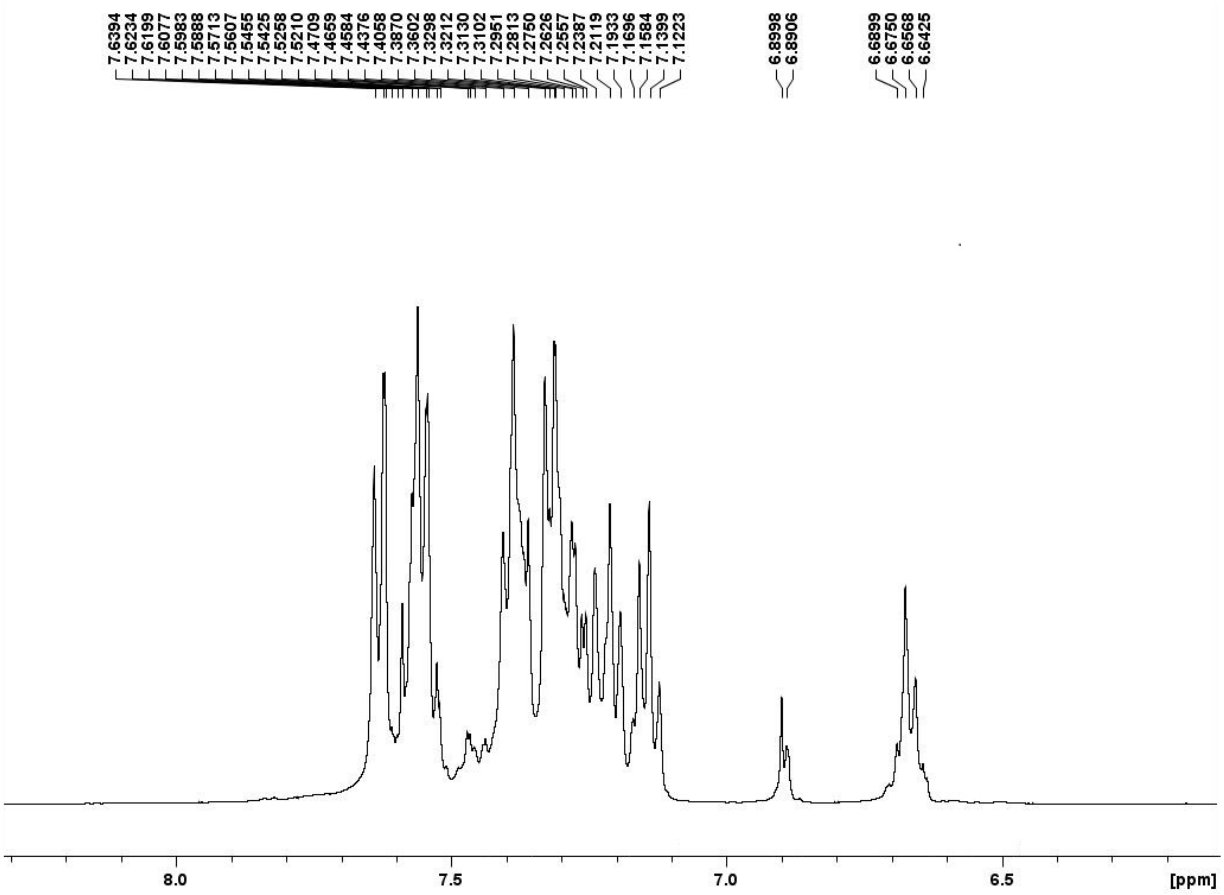

Fig. S13. Aromatic region of the ^{1}H NMR spectrum of [$Os_3(CO)_8(\eta^1$-Ph)($SbPh_3$)(μ-$SbPh_2$)(μ-dppm)] (**4**) in CD_2Cl_2.

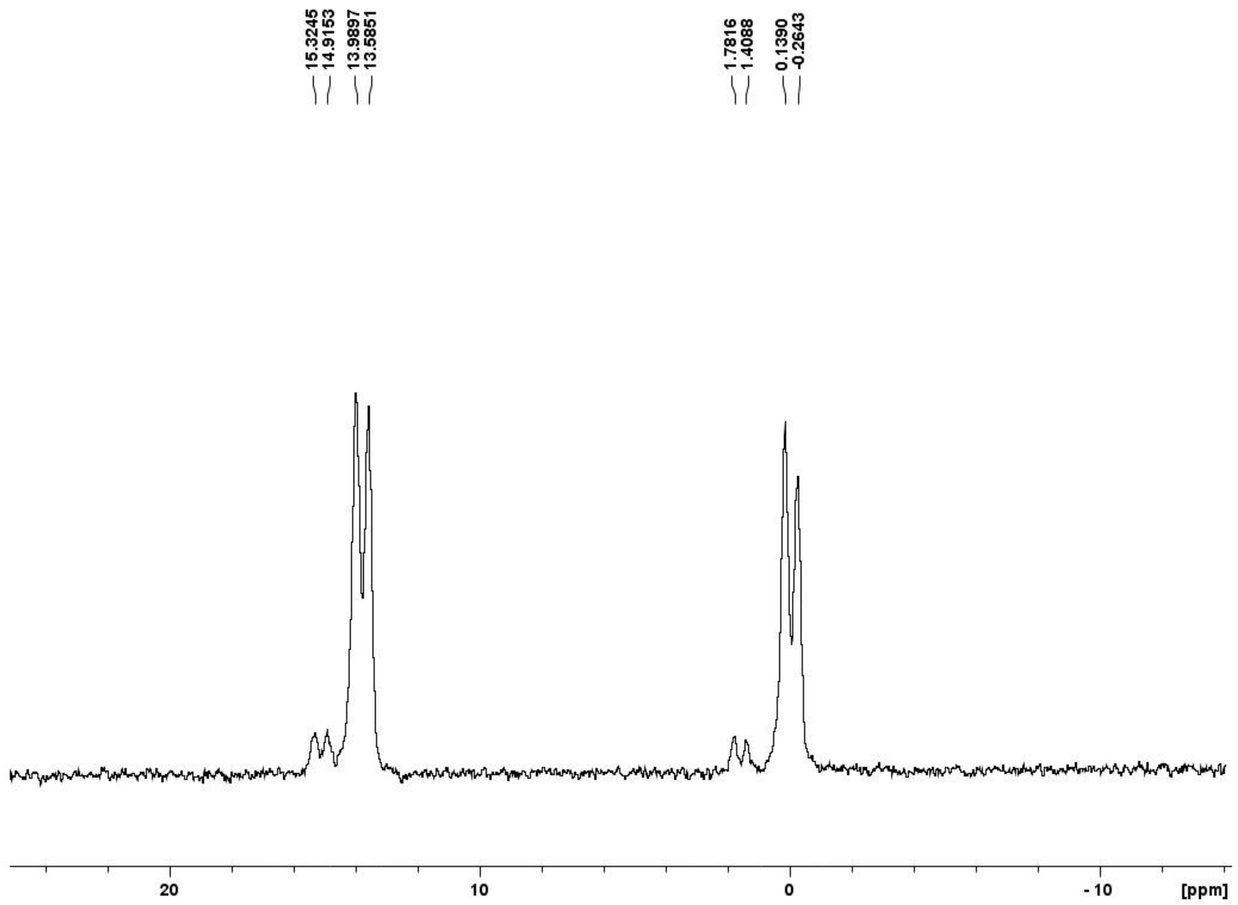

Fig. S14. $^{31}P\{^1H\}$ NMR spectrum of $[Os_3(CO)_8(\eta^1\text{-}Ph)(SbPh_3)(\mu\text{-}SbPh_2)(\mu\text{-}dppm)]$ (**4**) in CD_2Cl_2.

Table S1. Selected bond distances (Å) and angles (°) for clusters **1-4**

Cluster 1

Bond Distances

Os(1)−Os(2) 2.8807(2), Os(1)−Os(3) 2.9153(2), Os(2)−Os(3) 2.8648(2), Os(1)−P(1) 2.3363(9), Os(2)−P(2) 2.3234(9), Os(3)−Sb(1) 2.5921(3)

Bond Angles

Os(2)−Os(1)−Os(3) 59.240(5), Os(1)−Os(2)−Os(3) 60.982(5), Os(1)−Os(3)−Os(2) 59.779(5), Sb(1)−Os(3)−Os(1) 93.868(8), Sb(1)−Os(3)−Os(2) 153.206(9), P(1)−Os(1)−Os(2) 88.54(2), P(1)−Os(1)−Os(3) 147.54(2), P(2)−Os(2)−Os(1) 94.12(2), P(2)−Os(2)−Os(3) 153.06(2)

Cluster 2

Bond Distances

Os(1)−Os(2) 2.8249(3), Os(1)−Os(3) 2.7720(3), Os(2)−Os(3) 2.8101(3), Os(1)−P(1) 2.3290(12), Os(2)−P(2) 2.3314(12), Os(3)−Sb(1) 2.6343(4), Os(1)−C(10) 2.232(4), Os(3)−C(10) 2.399(4)

Bond Angles

Os(2)−Os(1)−Os(3) 60.266(7), Os(1)−Os(2)−Os(3) 58.935(7), Os(1)−Os(3)−Os(2) 60.799(6), Sb(1)−Os(3)−Os(1) 109.604(9), Sb(1)−Os(3)−Os(2) 169.035(10), Sb(1)−Os(3)−C(10) 93.54(10), P(1)−Os(1)−Os(2) 90.71(3), P(1)−Os(1)−Os(3) 131.53(3), P(2)−Os(2)−Os(1) 77.51(3), P(2)−Os(2)−Os(3) 85.60(3), C(10)−Os(1)−Os(3) 56.06(11), Os(1)−C(10)−Os(3) 73.42(13)

Cluster 3

Bond Distances

Os(1)−Os(2) 3.1729(4), Os(2)−Os(3) 2.9725(4), Os(1)−P(1) 2.335(2), Os(2)−P(2) 2.3328(19), Os(1)−Sb(1) 2.6400(6), Os(3)−Sb(1) 2.7081(5), Os(3)−Sb(2) 2.6137(6), Os(1)−C(8) 2.134(7), Os(2)−C(9) 2.128(7)

Bond Angles

Os(2)−Os(1)−Os(3) 88.959(11), P(1)−Os(1)−Os(2) 87.45(5), P(1)−Os(1)−C(8) 85.7(2), P(1)−Os(1)−Sb(1) 163.05(5), Sb(1)−Os(1)−Os(2) 78.881(14), Sb(1)−Os(1)−C(8) 79.5(2), P(2)−Os(2)−Os(1) 89.72(5), P(2)−Os(2)−Os(3) 176.04(6), P(2)−Os(2)−C(9) 82.9(2), C(9)−Os(2)−Os(3) 93.2(2), C(9)−Os(2)−Os(1) 66.1(2), Sb(2)−Os(3)−Os(2) 176.300(19), Sb(1)−Os(3)−Sb(2) 100.789(18), Sb(1)−Os(3)−Os(2) 81.559(13), Os(1)−Sb(1)−Os(3) 107.319(19)

Cluster 4

Bond Distances

Os(1)−Os(2) 3.0211(3), Os(2)−Os(3) 3.0024(3), Os(1)−P(1) 2.3177(11), Os(2)−P(2) 2.3096(12), Os(1)−Sb(1) 2.6479(3), Os(3)−Sb(1) 2.6695(4), Os(3)−Sb(2) 2.6468(4), Os(1)−C(9) 2.143(5)

Bond Angles

Os(2)−Os(1)−Os(3) 92.473(7), P(1)−Os(1)−Os(2) 94.12(3), P(1)−Os(1)−C(9) 93.11(12), P(1)−Os(1)−Sb(1) 167.76(3), Sb(1)−Os(1)−Os(2) 75.271(9), Sb(1)−Os(1)−C(9) 97.26(12), P(2)−Os(2)−Os(1) 85.14(3), P(2)−Os(2)−Os(3) 176.03(3), Sb(2)−Os(3)−Os(2) 95.815(9), Sb(1)−Os(3)−Sb(2) 167.637(11), Sb(1)−Os(3)−Os(2) 75.292(8), Os(1)−Sb(1)−Os(3) 109.790(11), C(9)−Os(1)−Os(2) 172.35(12)

Computational Details

M06 geometries for the optimized structures **A-D**

Coordinates: **A**

Os	26.06520000	8.07750000	9.03760000
Os	28.84330000	7.22870000	9.84490000
Os	26.96270000	8.19650000	11.89280000
Sb	24.71850000	9.40190000	12.60700000
P	26.71000000	7.61310000	6.80480000
P	29.20130000	6.15570000	7.76590000
C	26.39260000	10.00860000	9.11080000
C	24.22030000	8.40840000	8.70090000
C	25.74840000	6.17280000	9.30030000
C	29.22420000	9.07380000	9.32190000
C	30.60100000	7.02670000	10.55010000
C	28.32420000	5.51480000	10.64940000
C	27.86060000	9.92370000	11.65660000
C	27.91100000	7.96330000	13.51590000
C	26.09250000	6.45710000	12.16360000
O	26.48660000	11.15990000	9.10990000
O	23.10540000	8.55000000	8.42040000
O	25.48890000	5.04310000	9.34190000
O	29.54990000	10.14700000	9.03860000
O	31.69410000	6.92210000	10.91470000
O	28.13630000	4.48010000	11.11970000
O	28.38570000	10.94870000	11.64830000
O	28.48630000	7.79750000	14.50550000
O	25.55560000	5.47930000	12.45310000
C	27.68520000	6.04230000	6.70420000
H	27.91830000	5.74320000	5.67080000
H	27.04270000	5.26590000	7.14010000
C	27.70610000	8.74900000	5.76700000
C	28.08630000	10.00960000	6.22400000
H	27.77760000	10.34820000	7.20890000
C	28.88120000	10.83900000	5.43470000
H	29.17930000	11.81450000	5.81690000
C	29.29380000	10.41550000	4.17730000
H	29.92210000	11.05870000	3.56140000
C	28.90050000	9.16420000	3.70120000
H	29.22290000	8.82510000	2.71700000
C	28.10980000	8.33800000	4.48860000
H	27.79970000	7.36600000	4.09920000
C	25.25600000	7.27130000	5.74570000
C	24.68100000	8.32180000	5.02250000
H	25.18080000	9.29180000	4.98310000
C	23.46810000	8.14160000	4.36620000
H	23.02730000	8.96870000	3.81050000
C	22.81690000	6.91200000	4.42420000
H	21.86420000	6.77330000	3.91400000

C	23.38300000	5.86210000	5.14250000
H	22.87740000	4.89830000	5.19570000
C	24.59350000	6.04070000	5.80490000
H	25.00710000	5.21420000	6.38550000
C	30.46620000	6.74990000	6.57610000
C	31.19220000	7.91830000	6.80290000
H	31.01850000	8.50140000	7.70570000
C	32.13950000	8.34990000	5.87530000
H	32.69510000	9.26780000	6.06410000
C	32.36890000	7.61360000	4.71900000
H	33.10970000	7.95170000	3.99470000
C	31.65350000	6.43830000	4.48840000
H	31.83820000	5.85230000	3.58830000
C	30.71110000	6.00760000	5.41250000
H	30.16980000	5.07540000	5.23600000
C	29.70300000	4.40770000	7.99050000
C	31.05360000	4.15100000	8.26240000
H	31.77960000	4.96670000	8.23070000
C	31.47640000	2.86480000	8.57380000
H	32.52930000	2.67910000	8.78390000
C	30.55590000	1.81910000	8.62150000
H	30.88640000	0.81100000	8.86900000
C	29.21230000	2.06820000	8.36350000
H	28.48440000	1.25910000	8.41340000
C	28.78460000	3.35720000	8.05370000
H	27.71940000	3.53150000	7.89470000
C	22.84230000	8.41000000	12.36500000
C	22.72010000	7.27130000	11.56750000
H	23.59440000	6.86650000	11.05530000
C	21.48180000	6.65130000	11.41560000
H	21.39760000	5.76210000	10.79150000
C	20.36150000	7.17140000	12.05660000
H	19.39150000	6.68950000	11.93490000
C	20.47730000	8.30770000	12.85560000
H	19.60040000	8.71340000	13.36000000
C	21.71490000	8.92430000	13.01430000
H	21.79910000	9.80830000	13.65110000
C	24.55570000	9.94660000	14.67150000
C	25.01980000	9.04320000	15.63240000
H	25.47640000	8.09820000	15.32630000
C	24.90570000	9.33980000	16.98810000
H	25.27240000	8.63150000	17.73070000
C	24.33110000	10.54320000	17.39000000
H	24.24680000	10.77820000	18.45100000
C	23.86800000	11.44820000	16.43840000
H	23.42070000	12.39110000	16.75340000
C	23.97850000	11.15210000	15.08100000
H	23.61750000	11.86990000	14.34020000
C	24.29590000	11.27990000	11.69430000
C	23.25490000	11.40710000	10.77060000

H	22.59730000	10.56200000	10.55710000
C	23.05020000	12.61910000	10.11430000
H	22.23870000	12.71220000	9.39270000
C	23.87730000	13.70670000	10.38210000
H	23.71520000	14.65430000	9.86850000
C	24.91240000	13.58630000	11.30630000
H	25.56110000	14.43600000	11.51760000
C	25.12630000	12.37470000	11.95770000
H	25.94430000	12.29110000	12.67780000

Coordinates: **A_alt**

Os	26.27800000	7.92370000	9.23160000
Os	29.08930000	7.02110000	9.70630000
Os	27.38160000	7.91930000	11.99810000
Sb	25.89050000	5.73280000	12.80910000
P	26.69930000	7.67080000	6.90430000
P	29.28400000	6.13790000	7.51490000
C	26.60860000	9.83930000	9.51120000
C	24.40000000	8.24000000	9.20860000
C	26.04150000	5.99360000	9.23040000
C	29.44350000	8.88720000	9.21930000
C	30.87140000	6.74170000	10.32260000
C	28.50770000	5.28300000	10.37740000
C	28.46620000	9.46380000	11.73900000
C	28.37890000	7.54560000	13.58650000
C	25.85470000	8.94890000	12.56050000
O	26.70930000	10.97700000	9.68180000
O	23.24960000	8.36170000	9.14960000
O	25.86990000	4.85790000	9.07300000
O	29.73220000	9.96700000	8.92450000
O	31.96790000	6.58370000	10.65770000
O	28.23440000	4.20860000	10.70780000
O	29.09650000	10.42960000	11.74970000
O	28.95590000	7.36740000	14.57340000
O	24.98820000	9.64190000	12.87500000
C	27.68150000	6.12720000	6.57870000
H	27.82150000	5.93170000	5.50510000
H	27.08790000	5.30080000	6.98910000
C	27.60510000	8.90760000	5.90220000
C	27.94980000	10.15190000	6.42620000
H	27.65210000	10.41650000	7.43710000
C	28.69700000	11.05530000	5.67300000
H	28.97270000	12.01510000	6.10770000
C	29.09750000	10.72030000	4.38500000
H	29.69240000	11.41980000	3.79790000
C	28.73120000	9.49000000	3.83870000
H	29.03770000	9.22520000	2.82700000
C	27.98860000	8.59050000	4.59110000

H	27.70630000	7.63210000	4.15110000
C	25.16840000	7.38290000	5.93830000
C	24.59600000	8.43490000	5.21640000
H	25.12530000	9.38530000	5.12880000
C	23.35180000	8.27910000	4.61200000
H	22.91650000	9.10770000	4.05400000
C	22.66550000	7.07360000	4.72350000
H	21.69050000	6.95330000	4.25220000
C	23.22650000	6.02290000	5.44610000
H	22.69370000	5.07720000	5.54370000
C	24.46730000	6.17590000	6.05500000
H	24.87180000	5.34890000	6.64070000
C	30.45680000	6.83980000	6.28900000
C	31.25760000	7.94060000	6.58850000
H	31.20640000	8.39750000	7.57490000
C	32.13240000	8.45660000	5.63310000
H	32.74720000	9.32130000	5.88030000
C	32.22080000	7.86590000	4.37810000
H	32.90730000	8.26730000	3.63300000
C	31.43980000	6.74930000	4.07730000
H	31.51840000	6.27300000	3.10020000
C	30.56450000	6.23960000	5.02680000
H	29.97040000	5.35400000	4.78950000
C	29.80210000	4.38090000	7.53610000
C	31.16720000	4.12510000	7.72450000
H	31.87610000	4.95600000	7.75270000
C	31.62590000	2.82300000	7.87500000
H	32.69000000	2.63940000	8.02050000
C	30.72630000	1.75820000	7.84340000
H	31.08530000	0.73660000	7.96380000
C	29.36980000	2.00470000	7.66390000
H	28.65990000	1.17850000	7.64550000
C	28.90620000	3.31040000	7.51360000
H	27.83460000	3.47610000	7.39900000
C	26.83870000	4.10210000	13.81690000
C	28.22730000	3.95190000	13.80600000
H	28.85660000	4.63780000	13.23540000
C	28.82480000	2.92050000	14.52770000
H	29.90910000	2.81270000	14.51740000
C	28.03870000	2.03430000	15.25820000
H	28.50830000	1.22720000	15.82040000
C	26.65350000	2.18210000	15.27810000
H	26.03730000	1.49500000	15.85800000
C	26.05460000	3.21720000	14.56570000
H	24.96890000	3.33670000	14.60300000
C	24.67520000	6.37420000	14.46110000
C	25.30450000	6.99950000	15.54380000
H	26.38530000	7.16180000	15.54190000
C	24.56160000	7.42220000	16.64220000
H	25.06240000	7.90940000	17.47870000

C	23.18260000	7.22610000	16.66800000
H	22.59990000	7.56080000	17.52610000
C	22.55110000	6.59880000	15.59890000
H	21.47270000	6.43990000	15.61720000
C	23.29440000	6.17040000	14.49950000
H	22.78730000	5.67250000	13.67070000
C	24.37730000	4.75090000	11.67230000
C	24.44100000	3.38470000	11.39660000
H	25.24360000	2.77550000	11.81790000
C	23.49030000	2.79920000	10.56260000
H	23.54350000	1.73200000	10.34690000
C	22.48200000	3.57590000	9.99710000
H	21.74490000	3.11600000	9.33870000
C	22.41560000	4.94160000	10.26780000
H	21.63480000	5.55760000	9.82160000
C	23.36200000	5.52840000	11.10410000
H	23.31250000	6.60540000	11.29190000

Coordinates: **B**

Os	10.00500000	9.44020000	9.89590000
Os	12.09740000	11.35370000	10.56800000
Os	10.31290000	11.88000000	8.39480000
Sb	8.18770000	12.02250000	6.73830000
P	11.56090000	7.67980000	10.19590000
P	13.15040000	9.91920000	8.99990000
C	8.57540000	8.28970000	9.37270000
C	9.46830000	9.59230000	11.72570000
C	13.27330000	12.87090000	10.31060000
C	12.93030000	10.41950000	12.02050000
C	10.79710000	12.24670000	11.70210000
C	11.54740000	12.77270000	7.30230000
C	9.94090000	13.50610000	9.22080000
O	7.73800000	7.53640000	9.10170000
O	9.11950000	9.70800000	12.82240000
O	13.97780000	13.77030000	10.14500000
O	13.43240000	9.81870000	12.87440000
O	10.03380000	12.76350000	12.39210000
O	12.33970000	13.30250000	6.63750000
O	9.70800000	14.52190000	9.72430000
C	13.26730000	8.20080000	9.66920000
H	13.89520000	8.26490000	10.56940000
H	13.71960000	7.48240000	8.96930000
C	12.14400000	9.65760000	7.50670000
C	10.75700000	9.54220000	7.78210000
C	9.95890000	9.13280000	6.68040000
H	8.88300000	8.99240000	6.82160000
C	10.49000000	8.87650000	5.42330000
H	9.83210000	8.57100000	4.60820000

C	11.85990000	9.03720000	5.19540000
H	12.27490000	8.86020000	4.20240000
C	12.69390000	9.42130000	6.23880000
H	13.76770000	9.53030000	6.07710000
C	14.84860000	10.26140000	8.41070000
C	15.92550000	9.40350000	8.65310000
H	15.77440000	8.46100000	9.17920000
C	17.20890000	9.74560000	8.23200000
H	18.04010000	9.07030000	8.43350000
C	17.42670000	10.94280000	7.55860000
H	18.43110000	11.20990000	7.23100000
C	16.35870000	11.80290000	7.30870000
H	16.52130000	12.74530000	6.78700000
C	15.08000000	11.46910000	7.73800000
H	14.25490000	12.15740000	7.54640000
C	11.80520000	6.92920000	11.84720000
C	10.71290000	6.82500000	12.71330000
H	9.74150000	7.21540000	12.41060000
C	10.85150000	6.21760000	13.95760000
H	9.99190000	6.15320000	14.62350000
C	12.08550000	5.70810000	14.34950000
H	12.19990000	5.24560000	15.32940000
C	13.17420000	5.78630000	13.48440000
H	14.14040000	5.38010000	13.78200000
C	13.03340000	6.38560000	12.23770000
H	13.89290000	6.42130000	11.56720000
C	11.23270000	6.20760000	9.14510000
C	11.55810000	6.21760000	7.78470000
H	12.09220000	7.06290000	7.34740000
C	11.19450000	5.15600000	6.96340000
H	11.45560000	5.18470000	5.90510000
C	10.49910000	4.07060000	7.48960000
H	10.21280000	3.23890000	6.84640000
C	10.17220000	4.05180000	8.84250000
H	9.63080000	3.20540000	9.26420000
C	10.53510000	5.11320000	9.66630000
H	10.27310000	5.08740000	10.72520000
C	6.64980000	10.55020000	6.92860000
C	6.32200000	9.67690000	5.88750000
H	6.81440000	9.77060000	4.91570000
C	5.36600000	8.68220000	6.08430000
H	5.11650000	8.00130000	5.27050000
C	4.73010000	8.55980000	7.31690000
H	3.98330000	7.78090000	7.46890000
C	5.05290000	9.42580000	8.35900000
H	4.56630000	9.32220000	9.32860000
C	6.01910000	10.41020000	8.17050000
H	6.28760000	11.06590000	9.00370000
C	8.54310000	12.01380000	4.62950000
C	9.84350000	11.82990000	4.15380000

H	10.66670000	11.67420000	4.85540000
C	10.09710000	11.83260000	2.78370000
H	11.11500000	11.68720000	2.42050000
C	9.05160000	12.02460000	1.88470000
H	9.24870000	12.02690000	0.81270000
C	7.75360000	12.22300000	2.35270000
H	6.93800000	12.38340000	1.64760000
C	7.49920000	12.22230000	3.72160000
H	6.48110000	12.39100000	4.08230000
C	7.02050000	13.80900000	6.84620000
C	7.69310000	15.03230000	6.75520000
H	8.78170000	15.05980000	6.65260000
C	6.98070000	16.22750000	6.78740000
H	7.51070000	17.17730000	6.71890000
C	5.59340000	16.20690000	6.91110000
H	5.03580000	17.14280000	6.93990000
C	4.91810000	14.99230000	6.99700000
H	3.83230000	14.97580000	7.09060000
C	5.62860000	13.79370000	6.96360000
H	5.09150000	12.84480000	7.02850000
H	9.06670000	10.95150000	9.52930000

Coordinates: **C**

Os	10.40810000	15.99680000	7.87420000
Os	12.59910000	15.59510000	5.48510000
Os	10.30270000	15.43860000	3.53600000
Sb	8.85760000	15.09720000	5.87550000
Sb	8.21730000	15.10960000	1.89610000
P	12.26650000	16.35820000	9.29630000
P	14.31750000	15.47840000	7.12840000
C	9.21930000	15.41400000	9.23280000
C	9.78050000	17.83910000	7.94420000
C	13.63370000	14.69610000	4.19290000
C	12.92600000	17.37690000	4.77470000
C	9.99620000	17.31240000	4.01510000
C	11.60980000	15.92110000	2.22730000
C	10.83480000	13.55430000	3.45630000
O	8.50840000	15.05080000	10.06850000
O	9.42010000	18.93450000	8.02380000
O	14.26630000	14.16190000	3.38260000
O	13.07690000	18.46000000	4.39570000
O	9.82970000	18.40400000	4.34150000
O	12.42700000	16.23710000	1.47480000
O	11.15510000	12.45280000	3.35910000
C	11.14880000	13.96370000	7.60690000
C	12.00130000	13.76990000	6.49620000
C	12.48440000	12.47470000	6.23930000
H	13.14900000	12.29560000	5.38690000

C	12.16060000	11.39430000	7.06140000
H	12.55810000	10.40210000	6.83760000
C	11.34850000	11.59650000	8.17450000
H	11.08250000	10.76130000	8.82530000
C	10.83910000	12.87090000	8.43310000
H	10.17090000	12.99780000	9.29150000
C	13.69450000	15.28180000	8.85760000
H	14.49910000	15.35580000	9.60280000
H	13.28310000	14.26460000	8.88540000
C	11.92510000	15.93410000	11.03470000
C	11.19010000	16.84430000	11.80560000
H	10.94740000	17.82920000	11.39930000
C	10.75630000	16.49620000	13.07880000
H	10.18540000	17.21190000	13.66940000
C	11.03950000	15.23150000	13.59190000
H	10.69180000	14.95580000	14.58700000
C	11.75920000	14.31900000	12.82770000
H	11.97710000	13.32640000	13.22070000
C	12.20090000	14.66580000	11.55290000
H	12.74480000	13.92930000	10.95960000
C	13.02870000	18.02070000	9.35490000
C	12.92820000	18.85460000	8.23720000
H	12.31380000	18.55260000	7.38790000
C	13.63060000	20.05560000	8.18730000
H	13.55500000	20.68380000	7.30010000
C	14.42420000	20.44030000	9.26340000
H	14.97570000	21.37950000	9.22640000
C	14.51500000	19.62420000	10.39030000
H	15.13100000	19.92700000	11.23710000
C	13.82670000	18.41700000	10.43510000
H	13.91040000	17.77520000	11.31470000
C	15.43000000	14.02480000	6.96390000
C	15.26920000	12.85230000	7.70670000
H	14.50150000	12.77290000	8.47540000
C	16.07320000	11.74340000	7.45750000
H	15.92430000	10.83450000	8.04030000
C	17.04570000	11.79030000	6.46450000
H	17.66820000	10.91850000	6.26510000
C	17.22110000	12.95790000	5.72520000
H	17.98270000	13.00880000	4.94790000
C	16.42130000	14.06670000	5.97520000
H	16.57510000	14.97640000	5.39180000
C	15.55160000	16.84180000	7.21410000
C	16.24930000	17.14200000	8.38890000
H	16.05610000	16.58990000	9.30830000
C	17.18880000	18.16670000	8.41220000
H	17.70540000	18.40090000	9.34320000
C	17.46520000	18.89070000	7.25540000
H	18.19930000	19.69580000	7.27670000
C	16.80370000	18.57770000	6.07230000

H	17.01940000	19.12850000	5.15730000
C	15.85300000	17.56150000	6.05420000
H	15.33900000	17.32330000	5.12350000
C	6.94500000	16.10040000	5.71820000
C	6.86860000	17.46900000	6.00730000
H	7.75040000	18.01230000	6.34990000
C	5.66820000	18.16390000	5.86660000
H	5.63080000	19.22800000	6.10190000
C	4.52380000	17.49810000	5.43330000
H	3.58320000	18.03820000	5.32340000
C	4.58660000	16.13790000	5.14340000
H	3.69580000	15.60780000	4.80330000
C	5.78690000	15.44240000	5.28510000
H	5.80840000	14.37480000	5.05280000
C	8.12760000	13.14000000	6.36680000
C	7.04110000	13.02920000	7.24080000
H	6.52330000	13.92400000	7.59410000
C	6.60710000	11.77700000	7.67110000
H	5.75800000	11.70410000	8.35100000
C	7.26120000	10.62470000	7.24080000
H	6.92580000	9.64590000	7.58470000
C	8.35320000	10.72900000	6.38290000
H	8.89210000	9.83620000	6.06340000
C	8.78500000	11.98050000	5.94970000
H	9.66800000	12.03840000	5.31230000
C	7.05280000	16.80190000	1.31270000
C	6.59630000	17.67890000	2.30260000
H	6.84120000	17.51240000	3.35560000
C	5.81290000	18.77580000	1.95430000
H	5.45760000	19.45160000	2.73270000
C	5.48710000	19.00480000	0.61970000
H	4.87740000	19.86650000	0.34790000
C	5.94040000	18.13470000	-0.36880000
H	5.68740000	18.31500000	-1.41370000
C	6.72060000	17.03260000	-0.02530000
H	7.07230000	16.35410000	-0.80560000
C	6.64940000	13.76790000	2.45920000
C	6.98740000	12.58790000	3.12680000
H	8.03260000	12.34990000	3.33930000
C	5.99340000	11.70990000	3.55290000
H	6.26970000	10.80950000	4.10260000
C	4.65610000	12.00030000	3.29380000
H	3.87640000	11.31730000	3.63080000
C	4.31350000	13.16460000	2.60820000
H	3.26640000	13.39070000	2.40400000
C	5.30600000	14.05330000	2.19880000
H	5.02710000	14.97890000	1.68870000
C	8.67350000	14.26300000	-0.01640000
C	7.68690000	13.57810000	-0.73470000
H	6.68580000	13.45450000	-0.31390000

C	7.97680000	13.04250000	-1.98700000
H	7.20380000	12.50930000	-2.54040000
C	9.25360000	13.18240000	-2.52640000
H	9.48000000	12.75930000	-3.50500000
C	10.24100000	13.85780000	-1.81500000
H	11.24160000	13.96620000	-2.23300000
C	9.95240000	14.39740000	-0.56290000
H	10.73700000	14.92430000	-0.01700000
H	11.52390000	16.61190000	6.58270000

Coordinates: **D**

Os	2.81130000	3.04470000	4.47040000
Os	5.52120000	3.17770000	5.95710000
Os	5.23510000	0.15000000	6.71220000
Sb	3.44210000	0.44260000	4.70560000
Sb	6.97970000	0.49910000	8.73720000
P	2.33060000	5.32530000	4.72550000
P	5.39490000	5.46400000	5.37270000
C	1.75380000	2.65390000	6.06250000
C	3.82000000	3.36790000	2.83980000
C	4.32710000	3.51000000	7.45610000
C	7.26110000	3.38700000	6.69820000
C	6.18710000	2.57670000	4.21940000
C	3.82500000	0.57700000	8.01420000
C	4.99780000	-1.72800000	6.86650000
C	6.63920000	-0.05350000	5.35210000
O	1.07250000	2.40350000	6.95760000
O	4.36460000	3.56470000	1.83890000
O	3.65880000	3.79950000	8.36400000
O	8.34190000	3.64130000	7.04080000
O	6.62500000	2.22500000	3.20880000
O	3.03090000	0.74360000	8.83090000
O	4.85140000	-2.87430000	6.91800000
O	7.45660000	-0.24920000	4.56750000
C	1.08410000	2.59270000	3.28230000
C	-0.23160000	2.76460000	3.75490000
H	-0.40640000	3.18190000	4.75000000
C	-1.35120000	2.41160000	3.00190000
H	-2.34580000	2.55620000	3.42800000
C	-1.20840000	1.87420000	1.72610000
H	-2.08200000	1.59530000	1.13670000
C	0.07700000	1.70630000	1.21820000
H	0.22370000	1.28950000	0.22000000
C	6.45630000	6.33000000	7.83330000
H	6.06180000	5.39780000	8.24620000
C	1.19110000	2.05640000	1.98140000
H	2.17570000	1.88530000	1.53730000
C	3.73040000	6.25930000	5.52790000

H	3.77480000	7.31780000	5.22830000
H	3.54600000	6.25730000	6.61300000
C	0.91240000	5.71410000	5.82510000
C	1.03300000	5.63470000	7.21680000
H	1.98850000	5.39510000	7.68640000
C	-0.07970000	5.81550000	8.03170000
H	0.03210000	5.74770000	9.11330000
C	-1.32840000	6.06250000	7.46850000
H	-2.19980000	6.19760000	8.10860000
C	-1.46110000	6.12600000	6.08420000
H	-2.43630000	6.30830000	5.63320000
C	-0.34900000	5.95250000	5.26600000
H	-0.46540000	5.99750000	4.18230000
C	1.93930000	6.35210000	3.25440000
C	1.78060000	5.76040000	2.00090000
H	1.83430000	4.67610000	1.90610000
C	1.53370000	6.54830000	0.87760000
H	1.41370000	6.07230000	-0.09500000
C	1.43800000	7.92980000	1.00070000
H	1.25000000	8.54560000	0.12140000
C	1.56470000	8.52750000	2.25540000
H	1.46990000	9.60810000	2.36010000
C	1.80450000	7.74240000	3.37650000
H	1.88170000	8.21600000	4.35780000
C	6.33440000	6.60910000	6.46740000
C	6.86870000	7.80040000	5.96500000
H	6.78130000	8.02900000	4.90160000
C	7.51710000	8.69450000	6.81150000
H	7.93450000	9.61610000	6.40630000
C	7.63210000	8.41090000	8.16960000
H	8.14200000	9.10980000	8.83240000
C	7.09900000	7.22950000	8.67770000
H	7.19150000	6.99130000	9.73740000
C	6.01150000	5.90250000	3.71100000
C	5.30100000	6.70100000	2.81250000
H	4.33950000	7.13110000	3.08680000
C	5.80560000	6.94820000	1.53770000
H	5.22360000	7.55380000	0.84290000
C	7.03360000	6.41880000	1.15840000
H	7.42440000	6.60390000	0.15830000
C	7.76860000	5.65380000	2.06360000
H	8.73690000	5.24480000	1.77770000
C	7.26080000	5.39730000	3.33030000
H	7.83880000	4.79220000	4.03240000
C	1.70120000	-0.75030000	5.12740000
C	1.51160000	-1.44070000	6.32570000
H	2.28780000	-1.44510000	7.09290000
C	0.32580000	-2.13560000	6.56160000
H	0.19040000	-2.66940000	7.50250000
C	-0.68050000	-2.14210000	5.60050000

H	-1.60970000	-2.68060000	5.78760000
C	-0.49990000	-1.45600000	4.40150000
H	-1.28590000	-1.44640000	3.64580000
C	0.68440000	-0.76420000	4.16410000
H	0.80250000	-0.21790000	3.22480000
C	4.19440000	-0.67420000	3.03500000
C	4.25560000	-2.06910000	3.13400000
H	3.87870000	-2.57680000	4.02600000
C	4.79670000	-2.82200000	2.09560000
H	4.84080000	-3.90810000	2.17990000
C	5.28080000	-2.18790000	0.95270000
H	5.70700000	-2.77790000	0.14120000
C	5.22120000	-0.80150000	0.84880000
H	5.60080000	-0.30080000	-0.04190000
C	4.67960000	-0.04480000	1.88700000
H	4.65170000	1.04150000	1.79470000
C	6.69790000	-0.88480000	10.34560000
C	6.64110000	-0.46220000	11.67640000
H	6.72510000	0.59880000	11.92010000
C	6.47540000	-1.39440000	12.69920000
H	6.42800000	-1.05880000	13.73520000
C	6.37220000	-2.74990000	12.39910000
H	6.24190000	-3.47740000	13.20010000
C	6.43420000	-3.17810000	11.07490000
H	6.35430000	-4.23850000	10.83660000
C	6.59470000	-2.24890000	10.05070000
H	6.64050000	-2.59710000	9.01600000
C	9.07000000	0.23920000	8.39920000
C	9.88180000	-0.27040000	9.41820000
H	9.44700000	-0.56010000	10.37780000
C	11.25160000	-0.41250000	9.21140000
H	11.88120000	-0.81100000	10.00680000
C	11.81450000	-0.04850000	7.99030000
H	12.88650000	-0.16260000	7.83030000
C	11.01020000	0.45960000	6.97360000
H	11.44750000	0.74560000	6.01750000
C	9.64000000	0.60500000	7.17610000
H	9.02190000	1.00990000	6.37370000
C	6.97370000	2.31450000	9.86920000
C	5.76660000	2.79230000	10.38960000
H	4.83190000	2.25210000	10.22560000
C	5.73690000	3.98210000	11.11290000
H	4.78900000	4.35040000	11.50480000
C	6.91420000	4.69770000	11.32010000
H	6.89270000	5.62560000	11.89330000
C	8.11890000	4.22780000	10.80090000
H	9.03910000	4.79180000	10.95430000
C	8.15110000	3.03700000	10.07820000
H	9.09490000	2.68290000	9.65960000

Coordinates: **Species D_alt1**

Os	2.69700000	3.01520000	4.24720000
Os	5.44780000	3.18130000	5.93530000
Os	5.10360000	0.18760000	6.77370000
Sb	3.36270000	0.47510000	4.72140000
Sb	6.93360000	0.45730000	8.73800000
P	2.24350000	5.30220000	4.70930000
P	5.31210000	5.46580000	5.35280000
C	1.36030000	2.73980000	2.95680000
C	3.93480000	3.37370000	2.80860000
C	4.42230000	3.63440000	7.52490000
C	7.21140000	3.34280000	6.62830000
C	6.18710000	2.52490000	4.24110000
C	3.76150000	0.77290000	8.08570000
C	4.71150000	-1.65640000	7.00880000
C	6.48060000	-0.15530000	5.41100000
O	0.60190000	2.60940000	2.08780000
O	4.55670000	3.56520000	1.84860000
O	3.89500000	4.02010000	8.48410000
O	8.31130000	3.55700000	6.93200000
O	6.70920000	2.15710000	3.28000000
O	3.02580000	1.03910000	8.92960000
O	4.45590000	-2.77870000	7.11580000
O	7.27290000	-0.40810000	4.61810000
C	1.13790000	2.48050000	5.75510000
C	1.16690000	2.82210000	7.11610000
H	2.01920000	3.35830000	7.52230000
C	0.12690000	2.53130000	8.00060000
H	0.22180000	2.82840000	9.04700000
C	-1.01600000	1.87790000	7.55430000
H	-1.83280000	1.64880000	8.23970000
C	-1.09110000	1.52260000	6.21080000
H	-1.96950000	1.00140000	5.82520000
C	6.53370000	6.28140000	7.78440000
H	6.27950000	5.29740000	8.18280000
C	-0.03890000	1.81400000	5.34660000
H	-0.15630000	1.48260000	4.31270000
C	3.63010000	6.20070000	5.56100000
H	3.62700000	7.28330000	5.35560000
H	3.47070000	6.07890000	6.64320000
C	0.78550000	5.67810000	5.75300000
C	0.83880000	6.28470000	7.00800000
H	1.78690000	6.60900000	7.43750000
C	-0.32820000	6.47970000	7.74460000
H	-0.27110000	6.95050000	8.72580000
C	-1.55370000	6.06860000	7.23470000
H	-2.46370000	6.21180000	7.81660000
C	-1.61640000	5.47040000	5.97660000

H	-2.57110000	5.13470000	5.57330000
C	-0.45520000	5.27520000	5.24230000
H	-0.51100000	4.79330000	4.26360000
C	1.90420000	6.41950000	3.28250000
C	2.01950000	5.97840000	1.96460000
H	2.26880000	4.94000000	1.75690000
C	1.84150000	6.86480000	0.90370000
H	1.93970000	6.50290000	-0.11920000
C	1.54990000	8.20110000	1.15290000
H	1.41810000	8.89720000	0.32470000
C	1.41250000	8.64870000	2.46740000
H	1.16760000	9.69140000	2.66870000
C	1.57860000	7.76150000	3.52390000
H	1.45220000	8.11360000	4.55030000
C	6.24130000	6.61760000	6.45800000
C	6.60230000	7.88660000	5.98750000
H	6.39080000	8.16290000	4.95320000
C	7.24000000	8.79770000	6.82200000
H	7.51940000	9.77830000	6.43730000
C	7.52600000	8.45380000	8.14080000
H	8.03010000	9.16500000	8.79470000
C	7.17110000	7.19610000	8.61790000
H	7.39860000	6.90830000	9.64430000
C	5.91790000	5.96030000	3.70550000
C	5.22160000	6.82700000	2.86090000
H	4.26450000	7.25060000	3.16320000
C	5.73690000	7.15180000	1.60810000
H	5.16630000	7.80990000	0.95270000
C	6.96010000	6.63120000	1.20060000
H	7.35910000	6.87880000	0.21740000
C	7.67930000	5.79560000	2.05440000
H	8.64310000	5.39330000	1.74510000
C	7.16160000	5.46260000	3.29920000
H	7.72700000	4.80600000	3.96380000
C	1.68870000	-0.83040000	5.04810000
C	1.27070000	-1.19200000	6.32860000
H	1.83280000	-0.86880000	7.20720000
C	0.11470000	-1.94810000	6.50720000
H	-0.20630000	-2.21470000	7.51420000
C	-0.63300000	-2.34760000	5.40290000
H	-1.54040000	-2.93540000	5.54160000
C	-0.22200000	-1.99230000	4.11960000
H	-0.80590000	-2.30100000	3.25210000
C	0.93470000	-1.23700000	3.94230000
H	1.24070000	-0.95410000	2.93100000
C	4.25100000	-0.66360000	3.12260000
C	4.37430000	-2.05140000	3.26640000
H	3.94840000	-2.55660000	4.13700000
C	5.03250000	-2.80500000	2.29900000
H	5.12300000	-3.88460000	2.42170000

C	5.57500000	-2.17960000	1.17760000
H	6.09690000	-2.76840000	0.42310000
C	5.44550000	-0.80340000	1.02070000
H	5.86470000	-0.30860000	0.14450000
C	4.78310000	-0.04800000	1.98820000
H	4.70420000	1.03100000	1.85430000
C	6.65200000	-0.87420000	10.39000000
C	6.74910000	-0.42620000	11.71070000
H	6.95920000	0.62440000	11.92120000
C	6.57620000	-1.31940000	12.76620000
H	6.64860000	-0.96320000	13.79390000
C	6.31060000	-2.66150000	12.50920000
H	6.17310000	-3.35830000	13.33580000
C	6.21980000	-3.11570000	11.19540000
H	6.01240000	-4.16570000	10.99060000
C	6.38840000	-2.22510000	10.13860000
H	6.31480000	-2.59450000	9.11320000
C	8.98560000	0.07060000	8.30100000
C	9.79980000	-0.53230000	9.26540000
H	9.38200000	-0.83780000	10.22760000
C	11.14960000	-0.74860000	9.00060000
H	11.78070000	-1.22070000	9.75340000
C	11.69090000	-0.36360000	7.77620000
H	12.74740000	-0.53540000	7.57070000
C	10.88460000	0.23830000	6.81360000
H	11.30480000	0.54070000	5.85500000
C	9.53400000	0.45640000	7.07380000
H	8.91350000	0.93260000	6.31280000
C	7.11330000	2.28230000	9.83880000
C	5.96980000	2.84940000	10.41070000
H	4.99330000	2.37330000	10.29700000
C	6.06090000	4.03850000	11.12960000
H	5.16120000	4.47700000	11.56090000
C	7.29760000	4.66100000	11.28670000
H	7.37120000	5.58660000	11.85920000
C	8.43980000	4.10120000	10.71740000
H	9.40660000	4.59080000	10.83470000
C	8.35020000	2.91290000	9.99540000
H	9.24710000	2.48550000	9.54270000

Coordinates: **D_alt2**

Os	2.91860000	2.97030000	4.77150000
Os	5.63150000	3.34300000	6.20780000
Os	5.65540000	0.33760000	7.01600000
Sb	3.55160000	0.39360000	5.20980000
Sb	5.53200000	-2.22860000	7.71090000
P	2.39980000	5.25410000	4.72160000
P	5.46420000	5.54790000	5.38190000

C	1.87010000	2.74740000	6.39850000
C	3.95480000	3.06840000	3.12440000
C	4.39490000	3.81300000	7.63750000
C	7.29730000	3.63130000	7.09170000
C	6.41410000	2.65510000	4.55390000
C	4.34750000	0.98910000	8.32610000
C	7.12610000	0.73280000	8.18560000
C	6.81090000	-0.11310000	5.49630000
O	1.19960000	2.57730000	7.32120000
O	4.51290000	3.10620000	2.11330000
O	3.73660000	4.18890000	8.51760000
O	8.31410000	3.89290000	7.57930000
O	6.93170000	2.27490000	3.59220000
O	3.57920000	1.32450000	9.11570000
O	8.00940000	0.95660000	8.89260000
O	7.48240000	-0.43040000	4.61780000
C	1.21740000	2.33630000	3.62920000
C	-0.10780000	2.60980000	4.02200000
H	-0.29960000	3.20060000	4.92190000
C	-1.21200000	2.13500000	3.31430000
H	-2.21620000	2.37070000	3.67190000
C	-1.04100000	1.35720000	2.17170000
H	-1.90250000	0.97930000	1.62060000
C	0.25530000	1.07600000	1.74780000
H	0.42770000	0.46320000	0.86050000
C	6.44850000	6.71350000	7.74580000
H	6.09200000	5.80830000	8.23970000
C	1.35370000	1.56040000	2.45900000
H	2.34720000	1.28820000	2.09130000
C	3.77650000	6.31170000	5.40370000
H	3.81010000	7.32250000	4.96880000
H	3.57540000	6.44490000	6.47840000
C	0.96440000	5.77310000	5.74490000
C	1.05750000	5.82840000	7.13970000
H	2.00150000	5.62300000	7.64670000
C	-0.06480000	6.11290000	7.91060000
H	0.02690000	6.15170000	8.99550000
C	-1.29680000	6.33220000	7.30050000
H	-2.17560000	6.55220000	7.90610000
C	-1.40300000	6.26090000	5.91440000
H	-2.36530000	6.42090000	5.42840000
C	-0.28070000	5.98250000	5.14020000
H	-0.37540000	5.92110000	4.05540000
C	2.01040000	6.07360000	3.12450000
C	1.84910000	5.31680000	1.96280000
H	1.89180000	4.22900000	2.01830000
C	1.61720000	5.94670000	0.74100000
H	1.50060000	5.34390000	-0.15880000
C	1.53410000	7.33330000	0.67390000
H	1.36120000	7.82470000	-0.28350000

C	1.65560000	8.09550000	1.83640000
H	1.56880000	9.18080000	1.79200000
C	1.88340000	7.46820000	3.05490000
H	1.95840000	8.07060000	3.96290000
C	6.33460000	6.84460000	6.35830000
C	6.80660000	8.00960000	5.74340000
H	6.72290000	8.12560000	4.66140000
C	7.39010000	9.01880000	6.50320000
H	7.76130000	9.91840000	6.01250000
C	7.50210000	8.87820000	7.88430000
H	7.96380000	9.66730000	8.47730000
C	7.02780000	7.72590000	8.50470000
H	7.11640000	7.60580000	9.58390000
C	6.12730000	5.80920000	3.70190000
C	5.38000000	6.35810000	2.65860000
H	4.35560000	6.68960000	2.81760000
C	5.93060000	6.47720000	1.38450000
H	5.32540000	6.89120000	0.57820000
C	7.23700000	6.06630000	1.14780000
H	7.66580000	6.15310000	0.14980000
C	8.00280000	5.55070000	2.19310000
H	9.03170000	5.23860000	2.01790000
C	7.45250000	5.42320000	3.46140000
H	8.05840000	5.01710000	4.27520000
C	1.81770000	-0.61580000	5.99900000
C	1.80010000	-1.12410000	7.29830000
H	2.69640000	-1.06800000	7.92080000
C	0.64970000	-1.70440000	7.82700000
H	0.66310000	-2.10190000	8.84310000
C	-0.50660000	-1.77590000	7.05640000
H	-1.41020000	-2.22720000	7.46670000
C	-0.50930000	-1.25590000	5.76390000
H	-1.41470000	-1.28710000	5.15660000
C	0.64260000	-0.67690000	5.23760000
H	0.61390000	-0.26030000	4.22910000
C	3.85280000	-0.72370000	3.38800000
C	2.95960000	-1.72530000	2.99290000
H	2.14780000	-2.03330000	3.65440000
C	3.08780000	-2.33620000	1.74720000
H	2.37630000	-3.10810000	1.44960000
C	4.11090000	-1.95740000	0.88170000
H	4.20750000	-2.43200000	-0.09480000
C	5.00790000	-0.96540000	1.26850000
H	5.81210000	-0.65870000	0.59990000
C	4.87910000	-0.35000000	2.51220000
H	5.58560000	0.43700000	2.77880000
C	4.07140000	-3.57520000	6.93520000
C	3.20570000	-4.26760000	7.78540000
H	3.30360000	-4.17420000	8.86950000
C	2.19270000	-5.05940000	7.25050000

H	1.50840000	-5.58640000	7.91540000
C	2.04760000	-5.16810000	5.87000000
H	1.24880000	-5.78120000	5.45350000
C	2.92210000	-4.49570000	5.01970000
H	2.81430000	-4.58020000	3.93790000
C	3.92950000	-3.69390000	5.54980000
H	4.59050000	-3.14450000	4.87200000
C	7.31700000	-3.31050000	7.27100000
C	7.27110000	-4.66050000	6.91010000
H	6.31090000	-5.17510000	6.82720000
C	8.45180000	-5.35170000	6.64720000
H	8.41280000	-6.40280000	6.36130000
C	9.67840000	-4.69940000	6.74420000
H	10.60050000	-5.24110000	6.53400000
C	9.72940000	-3.35490000	7.10390000
H	10.68860000	-2.84290000	7.17810000
C	8.55160000	-2.66070000	7.36590000
H	8.60100000	-1.60440000	7.64200000
C	5.32010000	-2.64350000	9.79490000
C	4.51020000	-1.83130000	10.59500000
H	4.01100000	-0.95510000	10.17600000
C	4.33460000	-2.13180000	11.94410000
H	3.70360000	-1.49370000	12.56240000
C	4.96710000	-3.24170000	12.49840000
H	4.83200000	-3.47320000	13.55470000
C	5.77280000	-4.05520000	11.70530000
H	6.26920000	-4.92290000	12.13980000
C	5.94840000	-3.76000000	10.35570000
H	6.58240000	-4.40300000	9.74040000
